# Interplay of Gene Expression Noise and Ultrasensitive Dynamics Affects Bacterial Operon Organization

**DOI:** 10.1371/journal.pcbi.1002672

**Published:** 2012-08-30

**Authors:** J. Christian J Ray, Oleg A. Igoshin

**Affiliations:** 1 Department of Bioengineering, Rice University, Houston, Texas, United States of America; 2 Department of Systems Biology, The University of Texas MD Anderson Cancer Center, Houston, Texas, United States of America; University of Illinois at Urbana-Champaign, United States of America

## Abstract

Bacterial chromosomes are organized into polycistronic cotranscribed operons, but the evolutionary pressures maintaining them are unclear. We hypothesized that operons alter gene expression noise characteristics, resulting in selection for or against maintaining operons depending on network architecture. Mathematical models for 6 functional classes of network modules showed that three classes exhibited decreased noise and 3 exhibited increased noise with same-operon cotranscription of interacting proteins. Noise reduction was often associated with a decreased chance of reaching an ultrasensitive threshold. Stochastic simulations of the *lac* operon demonstrated that the predicted effects of transcriptional coupling hold for a complex network module. We employed bioinformatic analysis to find overrepresentation of noise-minimizing operon organization compared with randomized controls. Among constitutively expressed physically interacting protein pairs, higher coupling frequencies appeared at lower expression levels, where noise effects are expected to be dominant. Our results thereby suggest an important role for gene expression noise, in many cases interacting with an ultrasensitive switch, in maintaining or selecting for operons in bacterial chromosomes.

## Author Summary

In some species, most notably bacteria, chromosomal genes are arranged into clusters called operons. In operons, the process of transcription is physically coupled: a single pass of the RNA polymerase enzyme reading that region of the chromosome simultaneously produces messenger RNA encoding multiple proteins. So far, we do not have a satisfying explanation for what evolutionary forces have maintained operons on bacterial chromosomes. We hypothesized that different types of interactions between operon-coded proteins affect how strongly operons are selected for between two genes. The proposed mechanism for this effect is that operons correlate gene expression noise, changing how it manifests in the post-translational network depending on the type of protein interaction. Mathematical models demonstrate that operons reduce noise for some types of interactions but not others. We found that operon-dependent noise reduction has an underlying dependence on surprisingly high sensitivity of the network to the ratio of proteins from each gene. Databases of genetic information show that *E. coli* has operons more frequently than random if operons reduce noise for the type of interaction various gene pairs have, but not otherwise. Our study thus provides an example of how the architecture of post-translational networks affects bacterial evolution.

## Introduction

The organization of genes into operons is a prominent feature of bacterial chromosomes [1] that appear in some eukaryotes as well [2]. An operon is typically characterized as a promoter followed by multiple genes that are cotranscribed so that each transcription initiation event produces a polycistronic messenger RNA (mRNA) encoding multiple gene products [3]. Hypotheses explaining the emergence and maintenance of operons include proportional coregulation [4], [5], [6], [7], [8], [9], horizontal transfer of intact “selfish” operons [10], emergence via gene duplication [11], coproduction of physically interacting proteins to speed their association [12], [13], evolvability of co-regulation for interacting protein products [14], and reduction of intrinsic noise [15]. Current evidence favors some hypotheses more than others, but fails to indicate a definitive explanation for how operons are maintained in bacterial chromosomes [4], [11], [12], [13].

Arising from stochasticity of individual biochemical reactions and low copy numbers of reactants per cell, intrinsic noise plays a central role in network dynamics [16], [17]. In bacteria, intrinsic noise is most evident in gene expression, caused by translational bursting arising from small numbers of mRNA producing many proteins [18], [19] and transcriptional bursting arising from slow activation-deactivation cycles of transcriptional activity by unknown mechanism [20], [21]. Extrinsic noise, caused by uncertainties in global parameters and states including those characterizing transcriptional and translational machinery, also contributes to overall biochemical noise [22]. Noise in protein levels is commonly characterized by coefficient of variation (CV), the normalized root-mean square deviation of the protein levels from their mean value (CV = σ/μ, where σ is the standard deviation and μ is the mean) but other measures such as autocorrelation and covariance between concentrations of different proteins can give additional insights.

The effects of intrinsic noise on operon maintenance are not well characterized, but covariance between protein levels arising from intrinsic noise depends on transcriptional coupling (co-expression from an operon) of the corresponding genes [15]. The order of genes within an operon may also affect noise [23]. Therefore, we hypothesize that noise-related effects contribute to the evolutionary maintenance of operons. Studies of several specific systems corroborate that correlative effects of transcriptional coupling alter posttranslational dynamics [6], [24], [25]. However, it is still not clear how different classes of protein interactions and co-expression from an operon may interact to alter biochemical noise.

In this study we assessed these effects for different types of posttranslational interaction between gene products. In several types of interactions, the noise difference between cotranscribed and uncoupled configurations was amplified by the existence of a zero-order ultrasensitive switch [26]. We related the results to an intact naturally occurring system with simulations of cotranscribed and uncoupled configurations of the *lac* operon. To test our predictions bioinformatically, we classified naturally occurring interacting pairs of *E. coli* proteins by their type of interaction and analyzed the effect of chromosomal distance between pairs of genes with interacting products. Finally, we used single-cell protein copy number data to determine differences in operon frequencies at high and low expression levels in *E. coli* for physically interacting protein pairs.

## Results

### Operons correlate intrinsic protein fluctuations

**Figure 1:**
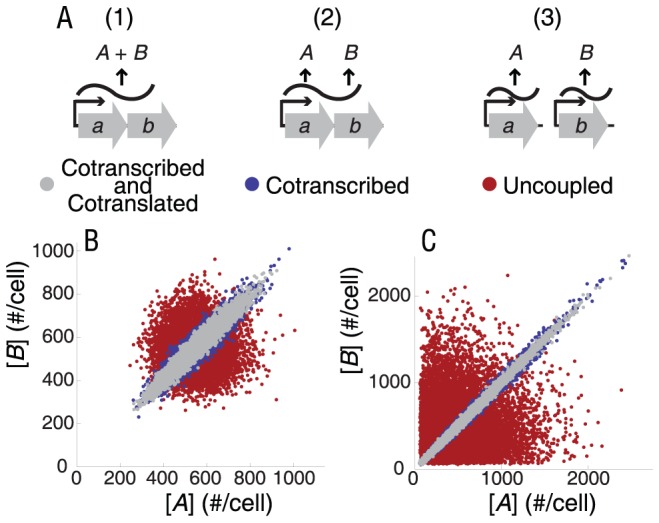
Types of coupling in protein production affect correlations in their fluctuations. A. Two proteins (*A* and *B*) can have coupled translation (1), coupled transcription with independent translation events (2), or uncoupled transcription (3). B, C. Scatter plots showing predicted single-cell distributions of copy numbers for proteins *A* and *B* with different coupling as indicated in models without (panel B) or with (panel C) transcriptional bursting. For simplicity, these models do not include extrinsic noise.

Intrinsic gene expression noise is correlated in a cotranscribed two-gene configuration, but this correlation was not seen in an uncoupled configuration. Relationships between the fluctuations of two proteins can be quantitatively characterized by the covariance of concentrations for proteins *A* and *B* (*σ_AB_*). Using the linear noise approximation (LNA; see Materials and Methods) [18], , we calculated a normalized covariance
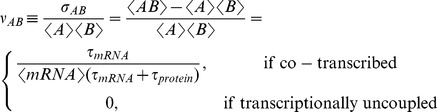
(1)where angle brackets represent average copy-number of each molecular species, and *τ_mRNA_* and *τ_protein_* are the characteristic timescales of mRNA and protein decay. Increased covariance of cotranscribed genes is preserved regardless of whether the translation processes are coupled (with a single ribosome binding site for multiple genes; Figure 1A–C) and regardless of the source of intrinsic noise (from translational bursting only or from both transcriptional and translational bursting; Figure 1B–C). We hypothesized that positive covariance can increase or decrease CV in relevant network outputs depending on the nature of the interactions between two proteins.

In addition to covariance, the effect of operons on correlation between protein copy number fluctuations can be quantified by another measure, the *degree of decorrelation* (Text S1). This measure is useful to characterize the effect of gene expression level on noise differences between cotranscribed and uncoupled proteins (Table S1), and to assess the effects of expression level on frequencies of operon occurrence in bacterial genomes.

### Effects of operons on biochemical noise depends on the type of protein interaction

**Figure 2:**
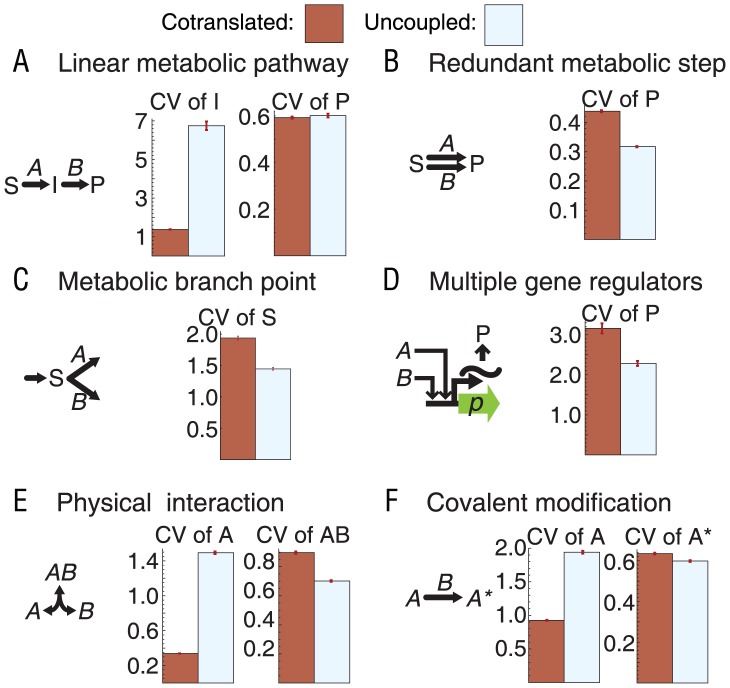
Noise levels in physiologically relevant variables depend on transcriptional coupling. We computed coefficient of variation [CV] = σ/μ, where σ is the standard deviation and μ is the mean, for simplified modules representing modes of interaction between two proteins: A) catalysis of subsequent steps in a linear metabolic pathway, B) redundant catalysis of the same metabolic step, C) catalysis of metabolic steps following a branch point, D) redundant regulation of a downstream gene *p* encoding protein *P*, E) physical interactions resulting in heterodimer formation, and F) covalent modification of one protein by another. In metabolic modules, S, I and P represent substrate, intermediate, and product, respectively. Complete reaction diagrams and parameters are given in supplemental tables (S4, S5, S6). Error bars represent one standard deviation from bootstrap resampling. Results correspond to the single ribosome binding site model (translational coupling), but hold qualitatively for multiple ribosome binding sites as well.

We surveyed databases of *E. coli* biochemical networks [29], [30], [31] to identify simple two-gene modules of larger networks that represent different ways that two proteins can directly or indirectly interact. The modules represent simple models of the following interactions: catalysis of subsequent steps in a linear metabolic pathway (Figure 2A), redundant catalysis of the same metabolic step (Figure 2B), catalysis of diverging reactions following a branch point in a metabolic pathway (Figure 2C), redundant transcriptional regulation of a downstream gene (Figure 2D), physical binding between two proteins (Figure 2E), and covalent modification of one protein by another (Figure 2F). The list may not be fully comprehensive, but represents several classes of interactions between proteins that are building blocks of larger networks. For each module we constructed a mathematical model to calculate CV for interacting proteins transcribed from the same and different operons (hereafter referred to as cotranscribed and uncoupled configurations, respectively). We then determined differences in CV for relevant network outputs between cotranscribed and uncoupled configurations. The simulations were controlled by keeping the same mean and CV for total protein from each gene between configurations. The CV calculations were performed at stationary state, both numerically (stochastic simulation algorithm; [32]) and analytically (LNA [28] using Paulsson's [18] normalization). The results of the simulations demonstrate that predicted differences in CV for each metabolic module depend on the type of interaction between proteins (Figure 2).

For the linear metabolic pathway module, cotranscription of two enzymes from the same operon results in lower CV for metabolic intermediate. Without transcriptional coupling, metabolic intermediate concentrations are prone to large spikes (Figures 2A and S1A, B, J, K). Notably, no significant differences between cotranscribed and uncoupled configurations are evident in metabolic product CV (Figure 2A), indicating that metabolic flux is not significantly different between the two groups. Intuitively, a spike occurs when flux from the upstream enzyme exceeds the maximal flux capacity of the downstream enzyme resulting in large increase of metabolic intermediate concentration. This increase exceeds the saturation point for the enzyme converting it to product, making product concentration insulated from these spikes.

In contrast, the metabolic modules with redundant enzymes (Figures 2B and S1C, L) and with a branch point (Figures 2C and S1D, M) show an increase in metabolite CV when the two enzymes are in the same operon. In these cases, lower correlations between enzyme fluctuations reduce the chance of simultaneous stochastic drops in concentration of both enzymes.

Similarly, cotranscription of multiple (redundant) gene regulators from the same operon results in increased CV in the regulated gene as compared to the uncoupled regulator configuration (Figures 2D and S1E, N). Here, we assumed that the gene regulatory logic was an OR gate (i.e., each regulator by itself or both together would have the same effect). Noise in the output from AND gate logic (i.e., a multi-subunit regulator) is expected to follow the noise pattern of the physical interaction module (below).

Consistent with a previous study [25], the physical protein interaction module under transcriptional coupling shows a strong reduction in fluctuations of monomer concentrations. (Figures 2E and S1F, G, O, P). With strong binding, the concentration of each free monomer changes from nearly zero when its partner is in excess to a finite value when the monomer itself is in excess. These fluctuations are more common when the binding partners are not in the same operon, so the noise is therefore high. Cotranscription slightly increases heterodimer CV compared to the uncoupled configuration (species *AB*; Figure 2E), but to a much lesser extent than its reduction of CV in monomer concentrations. In the limit of strong binding, nearly all of one monomer is bound, so the effect on monomer noise is dominant.

For the covalent modification module (Figures 2F and S1H, I, Q, R), different gene configurations cause small changes in the CV of the modified form of protein *A* (A*) that can be of either sign depending on parameter values, whereas the unmodified form (*A*) has consistently lower CV in the cotranscribed configuration (Figures 2F and S1H, Q).

### Analytical approach confirms predicted effects of cotranscription on intrinsic noise

The stochastic simulation approach (Figure 2) gives decisive results, but only for the parameter values tested. To determine how generally the simulation results hold in the face of different parameter values, we used the LNA to analytically determine noise differences (here quantified as CV^2^) between cotranscribed and uncoupled forms of each network module. For each molecular species denoted by *j*, we calculated the noise difference between cotranscribed (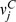) and uncoupled (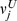) configurations as 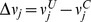. If the value is positive, the cotranscribed configuration has lower CV^2^ (and therefore, lower CV); if it is negative, the uncoupled configuration has lower CV^2^. A more complete analysis for each module is presented in Text S2. Here we highlight the main results.

1. Linear metabolic pathway: We find a lower bound on the difference in metabolic intermediate:
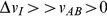
(2)Thus cotranscribed enzymes are generally predicted to have much lower noise in metabolic intermediate than uncoupled enzymes.
2. Redundant metabolic step: We find a difference in the product
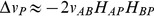
(3)where *H_AP_* and *H_BP_* are the logarithmic gains (sensitivities) of product *P* to proteins *A* and *B*, respectively. Because 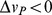, the uncoupled configuration is predicted to have lower noise.
3. Metabolic branch point: For substrate noise, we find
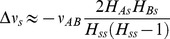
(4)where *H_As_*, *H_Bs_*, and *H_ss_* are the logarithmic gains of substrate flux ratio (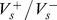, the ratio of the fluxes producing and consuming substrate) in response to changes in *A*, *B* and *s*, respectively. While *H_As_* and *H_Bs_* are positive, *H_ss_* is negative (Text S2). As the enzymes approach saturation, 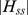 becomes small. As a result, 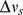 is large and positive, predicting much lower noise in the uncoupled configuration (Text S2).
4. Multiple gene regulators: The difference for protein production stimulated by two regulators with OR logic is
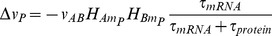
(5)where 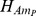 and 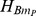 are the logarithmic gains of mRNA flux ratio (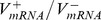, the ratio of mRNA production and degradation fluxes) in response to regulators *A* and *B*, respectively. Their absolute values are in the interval [0, 1] with positive values for activators and negative values for repressors. Thus, when both regulators are activators or repressors, 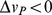, predicting more noise if regulators are in the same operon.
5. Physically interacting proteins: With strong interactions, we have
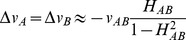
(6)where 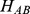 is the logarithmic gain of the flux ratio of *B* (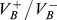, the ratio of fluxes producing unbound B to the fluxes consuming it) to protein *A*. We show that 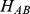 is negative with its absolute value approaching 1 from below. Therefore, 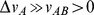, predicting that monomer noise is significantly decreased by covariance. At the same time, the difference in noise in heterodimer is negative and limited in absolute value to 
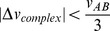
, predicting that complex noise slightly increases with cotranscription.
6. Covalent modification module: We have
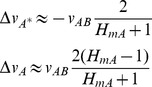
(7)where *H_mA_* represents the logarithmic gain of 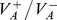 (the ratio of flux producing unmodified protein *A* to that consuming it) to concentration of mRNA. For realistic parameter values at which protein modification flux significantly exceeds the degradation flux, 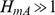 (Text S1). In this regime, 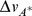 is small and negative whereas 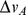 remains positive and significant, predicting a lower noise in the cotranscribed configuration.

To summarize, we found that differences in noise between cotranscribed and uncoupled configurations in stochastic simulations are qualitatively consistent with the analytical approach. Notably, in all the cases the magnitude of the differences in CV^2^ between two configurations is proportional to the value of covariance 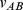, but in many cases the coefficient of proportionality is very large. This qualitatively suggests posttranslational interactions in some modules are capable of amplifying noise differences between cotranscribed and uncoupled proteins. However, the LNA method likely underestimates the magnitude of non-linear amplification. We further explore these amplification mechanisms in the next section.

### Ultrasensitivity arising in non-redundant protein interactions

**Figure 3:**
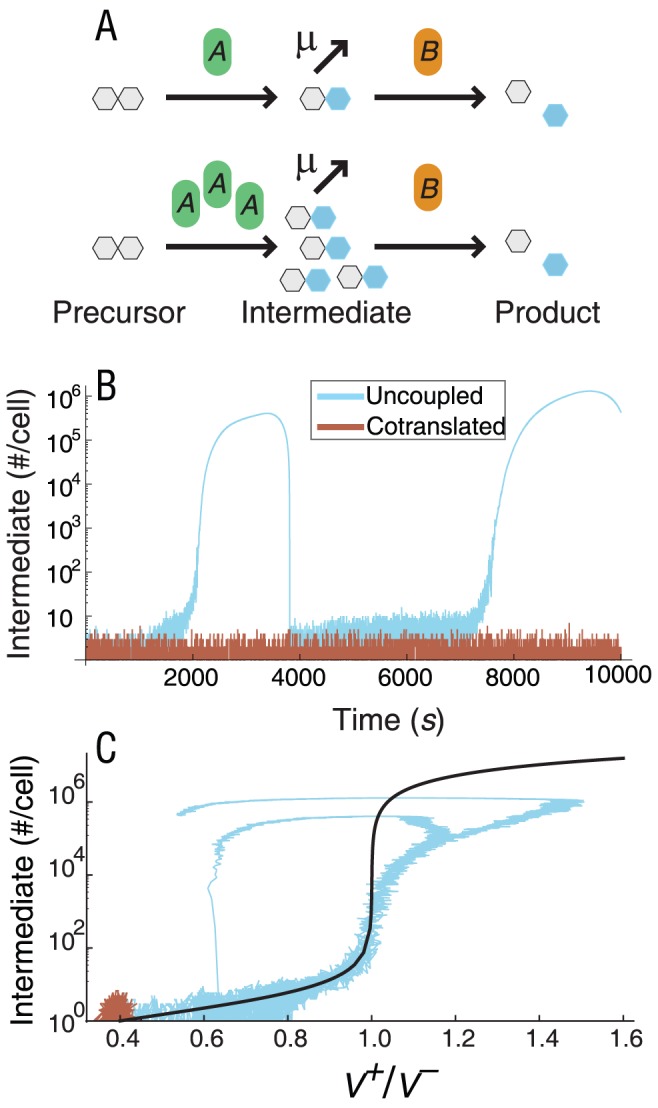
An ultrasensitive response amplifies noise differences between cotranscribed and uncoupled linear metabolic pathway modules. A. When copy numbers of enzymes A and B are matched, transient changes in production and consumption flux are matched, resulting in maintenance of a low concentration of metabolic intermediate. An increase in expression of *A*, unmatched by a change in expression of B, can cause the production flux of metabolic intermediate to exceed the saturation point of flux through enzyme *B*, resulting in accumulation of metabolic intermediate. B. Simulated timecourse of metabolic intermediate in cotranscribed and uncoupled configurations of the linear metabolic pathway model. C. Steady state response of metabolic intermediate to changes in the ratio of production flux to consumption flux (solid line), with stochastic simulation timecourses of intermediate in cotranscribed and uncoupled linear metabolic pathway module configurations plotted with respect to changing flux balance. Results represent the single ribosome binding site model (translational coupling), but are qualitatively the same for multiple ribosome binding sites as well.

Timecourse simulations predict that uncorrelated fluctuations of the two enzymes in a linear metabolic pathway result in large bursts of metabolic intermediate (Figure 3A, B). This suggests that higher noise in the transcriptionally uncoupled linear metabolic pathway arises at least in part from the increased probability of occasionally crossing an ultrasensitive threshold. Indeed, a sharp threshold in the intermediate of the linear metabolic pathway arises when the enzyme-mediated consumption of a product saturates, leading to non-linear degradation [33]. The ultrasensitive threshold is crossed when the downstream enzyme saturates, and the flux from the upstream enzyme exceeds its maximal value (*V^+^*/*V^−^*>1 in Figure 3C). Because *V^+^* and *V^−^* are proportional to their enzyme levels, the numerator and denominator of the ratio fluctuate together when both enzymes are in the same operon. Therefore transcriptional coupling lowers noise in the flux ratio and making it unlikely to cross the threshold *V^+^*/*V^−^* = 1. When the enzymes are uncoupled, simulations show more variability in the *V^+^*/*V^−^* ratio, allowing the ratio to cross the threshold with consequent large spikes in metabolic intermediate. Thus, the ultrasensitive switch amplifies noise differences already present between cotranscribed and uncoupled configurations.

Differences in noise between cotranscribed and uncoupled configurations of all of the non-redundant modules can be amplified by ultrasensitive switches in a similar manner (Figure S2). The metabolic branch point module undergoes the same type of non-linear degradation effect as the linear metabolic pathway, but in the branch point transcriptionally coupled enzyme pairs are more likely to fluctuate downward and saturate simultaneously than uncoupled enzymes. This effect leads to a higher likelihood of substrate buildup in the cotranscribed configuration (Figure S2B). The physical interaction and covalent modification modules undergo molecular titration [34], resulting in an ultrasensitive switch for monomers (physical interaction module) or unmodified protein (covalent module) that depends on the ratio of protein production fluxes (Figure S2C, D). Cotranscription of the two genes prevents the switch from amplifying transcriptional noise by reducing fluctuations in this ratio. Sensitivity analysis of mean-field models shows that the existence of ultrasensitive switches does not depend on strict parameter regimes (Text S3).

### Detailed *lac* operon model confirms effects of operons on intrinsic noise in an intact system

**Figure 4:**
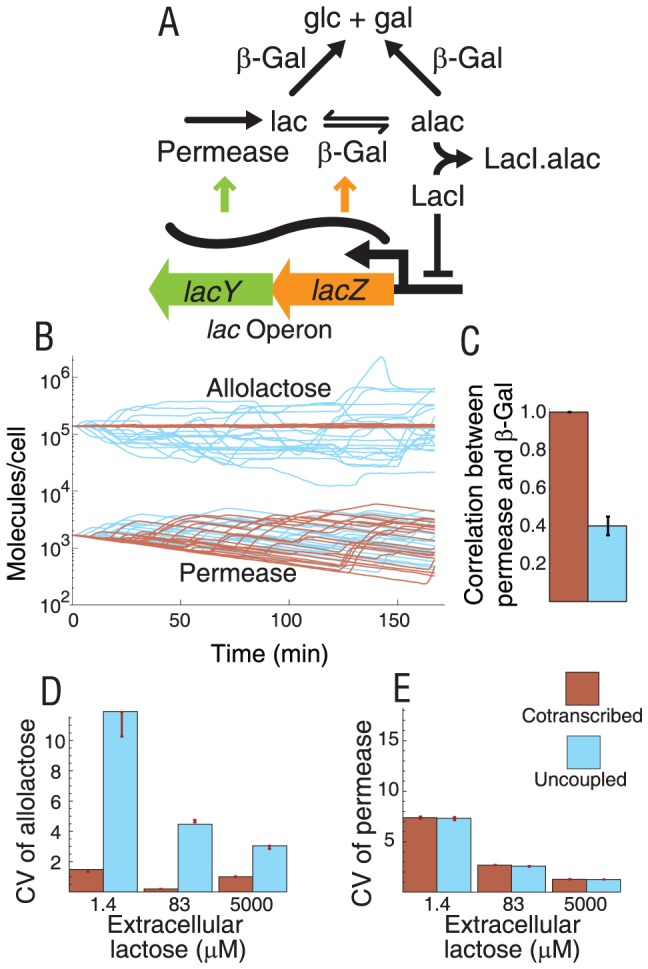
Allolactose noise depends on transcriptional coupling in simulations of the *E. coli lac* operon. A. Simplified reaction diagram of the model used. The complete reaction scheme and parameter values are given in Tables S7 and S8. Arrows depict flux arising from the mechanisms in the model. B. Predicted dynamics of allolactose (Alac) and permease (LacY) in excess inducer (extracellular lactose at 5,000 µM) are represented by 20 individual timecourses. C. Time correlation between permease and β-galactosidase (LacZ) for 100 timecourses. Error bars represent standard error of the mean. D. Coefficients of variation (CV = σ/μ, where σ is the standard deviation and μ is the mean) of metabolite allolactose at three extracellular lactose concentrations. E. CV of permease protein at three extracellular lactose concentrations. Bootstrapped mean and standard deviation of CVs (red bars in panels D and E) diverge from simulated CV when the distribution is highly skewed. Concentration 1.39 µM represents minimal induction of the *lac* operon; 83.0 µM represents mid-range induction; and 5,000 µM represents an excess of inducer with maximal *lac* operon induction. Error bars represent one standard deviation from bootstrap resampling.

To explore how conclusions drawn from models of simple network modules apply to a more complicated realistic network, we implemented stochastic simulations of a detailed *lac* operon model that is based on a previous deterministic model [35]. The stochastic model includes enzymatic steps reminiscent of a linear metabolic pathway with permease-mediated lactose import and conversion by β-galactosidase to allolactose and β-d-galactose+β-d-glucose (Figure 4A, Tables S7 and S8). Feedback and gene regulation are present with derepression of *lacY* and *lacZ* expression caused by allolactose binding to LacI (Figure 4A, Tables S7 and S8).

We simulated three inducer concentrations representing minimal *lac* operon induction (1.39 µM extracellular lactose concentration or 835 molecules/femtoliter), intermediate induction (83.0 µM or 50,000 molecules/femtoliter), and excess inducer with maximal *lac* operon induction (∼5,000 µM or 3×10^6^ molecules/femtoliter). Timecourses suggest that transcriptional coupling between *lacY* and *lacZ* (wild-type situation) eliminates the large fluctuations in allolactose (Figures 4B and S3) and intracellular lactose (not shown) observed in the transcriptionally uncoupled form of the system. This is consistent with a reduction in the correlation between permease and β-galactosidase (*lacY* and *lacZ* gene products, respectively) in time (Figure 4C).

At all inducer concentrations, the uncoupled configuration displays higher CV in allolactose than did the cotranscribed configuration (Figure 4D). This difference is most pronounced in the minimal induction region and gradually reduced with increasing *lacY-lacZ* induction. At the same time, there is little difference in protein CV between cotranscribed and uncoupled configurations of the model at most inducer levels. In both configurations the CV monotonically decreases with higher expression.

The primary consequence of cotranscription of *lac* proteins in the same operon is a reduction in fluctuations of intracellular lactose and allolactose. These fluctuations may prevent disruption of other sugar uptake pathways by, for example, interfering with inducer exclusion mechanisms [36]. Physiological benefits of noise reduction are also consistent with reports that excessive lactose import is associated with significant lowering of growth rate in *E. coli* [37 and references therein,38]. Thus, there may be a selective pressure to maintain high covariance between permease and β-galactosidase resulting from the wild-type genetic structure of the *lac* operon.

### Operon incidence in *E. coli* is correlated with noise reduction

To determine if global operon organization in *E. coli* correlates with predicted noise differences, we characterized frequencies of gene membership in the same operon bioinformatically (Table 1). We first assigned membership of known *E. coli* K12 MG1655 [39] biochemical networks into patterns corresponding to the 2-gene modules (Figure 2) using data on *E. coli* operons [31], metabolic pathways [29], gene regulation networks [31], covalent modification [30], and physical protein interactions [40], [41]. Many natural networks fall into more than one class (e.g., common bacterial signal mediators, two-component systems, have physical interactions between the sensor and the regulator [42] and are also in the covalent modification class). For the metabolic and gene regulation network modules, we eliminated physically interacting pairs to ensure that those included had true functional overlap and were not acting as subunits of a larger enzyme or regulator. Thus, the only systems that are members of more than one class are in members of both the covalent modification and physical interaction modules. In each class we created controls with randomized operon assignment of the genes (see Materials and Methods).

**Table 1:** Operon organization trends in *E. coli* relate to noise-minimizing transcriptional coupling patterns.

	Network module	Low noise configuration	Number of pairs	*f* a	*f*_rand_ (μ ± σ)	Trend in *E. coli* b
A.	Linear metabolic pathway	Coupled	2471	0.038	0.0036±0.0011	Coupled (<<10^−6^)
B.	Redundant enzymes	Uncoupled	114	0	0.01±0.00094	Uncoupled (<<10^−6^)
C.	Metabolic branch point	Uncoupled	2036	0.0025	0.0047±0.0015	Uncoupled (0.071)
D.	Multiple gene regulators	Uncoupled	1368	0.0015	0.043±0.0054	Uncoupled (<<10^−6^)
E.	Physical interaction	Coupled	3938	0.35	0.0030±0.00073	Coupled (<<10^−6^)
F.	Covalent modification	Coupled	201	0.23	0.0047±0.0047	Coupled (<<10^−6^)

a Same operon pair fraction.

b Numbers represent *p*-value vs rand.

Proteins in the linear metabolic pathway, physical interaction, and covalent modification modules appear in the same operon significantly more frequently than do randomized controls (*p*<<10^−6^; Table 1). On the other hand, redundant metabolic nodes and multiple gene regulators are significantly less likely to be in the same operon than randomized controls (*p*<<10^−6^; Table 1). Metabolic branch points show a bias toward being uncoupled, but falls just short of being statistically significant (*p* = 0.071). These findings hold even after we divide each class into essential and nonessential genes using data from Taniguchi *et al* [43]; Table S2). Thus operon overrepresentation, when it occurs, is present in essential genes, consistent with previous results contradicting the selfish operon hypothesis [12]. Our results establish a correlation between operon organization of protein pairs and their function that is consistent with noise minimization and avoidance of ultrasensitivity.

### Higher incidence of cotranscribed interacting proteins at low than high expression levels

To separate the specific effect of noise from that of other factors affecting selection for operons, such as proportional coregulation, we considered whether the tendency toward operon membership of posttranslationally interacting protein pairs is related to gene expression levels [23]. Intrinsic noise is stronger for genes with low expression levels [43], covariance of protein concentrations is more pronounced (Equation 1) and the degree of decorrelation is higher (Table S1). Therefore, if noise is an evolutionary factor driving operon formation, levels of gene expression may be inversely correlated with operon patterns. On the other hand, if coregulation of mean expression levels is the dominant factor in selecting for operons, the frequency of transcriptional coupling may be directly correlated with gene expression levels because the cost of differential regulation would be highest at the highest expression levels. As a result, any trend in coupling frequencies with gene expression levels would favor one hypothesis and disfavor the other.

We used a dataset of average single-cell mRNA and protein copy numbers in *E. coli* [43] to explore this trend for constitutively expressed physically interacting protein pairs (other network modules have insufficient data for such analysis). Because different conditions can shift gene expression levels and the dataset is only available for one condition, we chose to focus on the subset of interacting proteins that are constitutive, i.e., not predicted to undergo any regulation in RegulonDB. Each gene's protein or mRNA copy number was considered once, along with a binary variable indicating whether or not the protein product interacts with a same or non-same operon protein. Further details are given in Materials and Methods.

**Figure 5:**
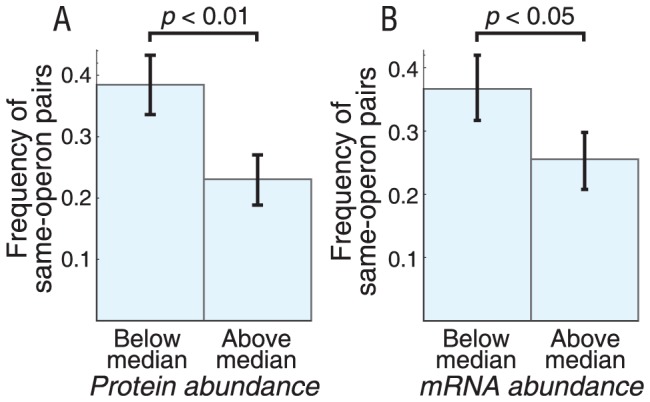
Dependence on expression level for frequencies of gene pairs of physically interacting proteins sharing operons. A dataset of single-cell protein and mRNA copy numbers in *E. coli* [43] shows reduced frequency of coupling for abundant proteins (A) and mRNA (B). Error bars represent one standard deviation from bootstrapping the data 1,000 times. Significance levels were determined by a bootstrap test.

We divided the set into two subsets of expression level, one below and one above the median copy number (Figure 5). The fraction of protein pairs sharing the same operon is higher in the low-expression subset for protein (bootstrap test *p*<0.01) and mRNA (bootstrap test *p*<0.05) copy numbers. This suggests that evolutionary selection against decorrelation (Table S1) significantly contributes to maintenance of operons in the chromosome.

## Discussion

A longstanding question in evolutionary biology is how non-transcriptional dynamics [44] affect selection of particular genetic architectures. By relating chromosomal patterns to protein network structures in *E. coli*, we see a compelling case for post-translational dynamics altering the probability of operon membership of genes depending on the nature of their interaction.

### Covariance and noise reduction in non-redundant interactions

Because enzymes often operate close to saturation [45], resolving metabolic flux imbalances may prevent widespread accumulation of intermediate, which is potentially toxic [46], [47], [48]. Simulations of a detailed *lac* operon model in our study corroborate the results of the simpler linear metabolic module, suggesting a role for intrinsic noise in selecting for *lac* operon architecture (in addition to the stochastic effects previously examined in this system [49]). Simulations that include extrinsic noise as a correlating factor indicate that it does not reduce metabolite noise as well as the stronger correlations caused by cotranscription (Text S4, Figure S4).

Many metabolic operons are large (and with complex evolutionary histories; [50]), but the length of a metabolic pathway is often longer than that of a typical operon, leading to the question of where optimal operon break points for metabolic pathways may lie. Our results suggest that break points occur predominantly where the intermediate is not toxic or where it is processed by multiple downstream enzymes, such as at branch points and metabolic steps with redundant enzymes. Metabolite spikes could also potentially be buffered by reversibility of catalytic reactions, though the reversible step in the *lac* operon did not prevent intermediate spikes. Furthermore, if portions of metabolic pathways that are divided by intermediates with relatively low toxicity undergo upregulation as needed, there may be a trade-off between reduction of toxic intermediate spikes and just-in-time transcription [51] in the evolution of metabolic networks.

Our analysis suggests that pairs of enzymes after a branch point can have lower noise (CV) if they are not cotranscribed (Figure 2C), but with a less consistent CV difference between cotranscribed and uncoupled configurations than the other modules (Figure S1 D,M). Therefore, the noise hypothesis predicts patterns of transcriptional coupling to be weaker than in other modules, as we observe to be the case in *E. coli* (Table 1).

The simple physical protein interaction module in our study (Figure 2E) may result in one of two different types of physiologically meaningful output variables: an active heterodimer, in which the genes make up subunits of a functional complex, or an active monomer, in which its activity is negatively regulated by the binding partner (as with sigma-antisigma systems [52]). In either case reduction in monomer noise is justified; in the latter case, to reduce noise in the physiologically relevant output. In the former case, lower noise represents a reduction in inefficient protein production that can reduce promiscuous interactions with other parts of the network. Heterodimer noise is smaller for the uncoupled configuration because upward fluctuations in its concentration are limited to being no larger than the minimum of [*A*] and [*B*] and those concentrations are less likely to simultaneously fluctuate upward simultaneously.

The covalent modification system (Figure 2F) in its uncoupled configuration has reduced fluctuations in the unmodified protein (*A*) compared with the uncoupled configuration. Noise effects of transcriptional coupling may therefore be important in covalent modification systems where the unmodified form of the protein is capable of interacting with other systems (Text S2).

Higher-order chromosome structure, such as bacterial chromatin [53], [54] and regulatory factors such as bidirectional promoters and transcriptional terminators [55] affect the spatial proximity of genes. Operons could also play a role in spatial proximity, as suggested by the selfish operon hypothesis [10]. We explored whether chromosomal proximity can explain operon membership in linear metabolic and physically interacting gene pairs. Our bioinformatic analysis suggests that the prevalence of operons cannot be solely explained by a proximity bias of interacting gene pairs in the *E. coli* chromosome (Text S4, Figure S5).

### Noise differences in non-redundant modules arise from ultrasensitivity

A striking feature of the non-redundant protein interaction modules is that they all contain a zero-order ultrasensitive switch, which arises as a side-effect of saturation. This effect amplifies differences in CV between cotranscribed and uncoupled forms of the modules (Figures 3 and S2) and may degrade performance when its threshold is crossed. In each two-gene module, the operon architecture that avoids crossing the ultrasensitive threshold is significantly over-represented in *E. coli* (Table 1). Signatures of selection against noise in these modules thus likely represent selection against performance-degrading ultrasensitivity as well.

### Gene expression level and operon prevalence

Gene pairs encoding constitutive physically interacting proteins are significantly more likely to be in the same operon if their expression levels are low (Figure 5). This trend could be explained by slow protein diffusion in the crowded intracellular environment, as cotranscribed gene products are more likely to be present at the same subcellular location. However even slow diffusion (<1 µm^2^/s) across a typical bacterial length of ∼1 µm is much faster than the expected time lag between translation of two proteins given typical ribosomal speeds of 12–21 AA/s [56]. Therefore, increased biochemical noise (here, measured as decorrelations between uncoupled proteins) at low expression levels are the most likely explanation of the observed trend. We argue that these noise effects are detrimental to the performance of some protein interaction networks.

The opposite trend would be expected if proportional expression of mean concentrations or other mechanisms are the primary selective pressure on operon maintenance. In general, genes with high expression levels may operate under greater evolutionary pressure than genes with low expression levels [57], [58] and therefore their deviation from optimal chromosomal organization is less likely. Arguably, noise minimization is the only selective force that is expected to be more important for genes with low expression levels than for genes with high expression levels [23].

### Redundant proteins and selection against operons

Partial functional redundancy of proteins allows one protein to compensate for a downward fluctuation in concentration of the other protein, thereby reducing noise with uncorrelated protein fluctuations (Table 1; Figure 2). Therefore, just as noise minimization may explain operon membership for non-redundant interactions, it may also explain the lack of redundant proteins in operons. Differential regulation of the genes can additionally play an important role in keeping redundant interactions transcriptionally uncoupled. In yeast metabolic pathways, apparently redundant enzymes are differentially expressed in different pathways depending on external conditions [59]. This type of mechanism, if present *in E. coli*, may also explain why no redundant enzymes are in the same operon. Similarly, different growth conditions may result in different regulators affecting downstream expression of the same genes. Further work is necessary to distinguish the noise reduction hypothesis more decisively from differential gene regulation as a selective force in redundant pairs; differential regulation may be physiologically important in some cases and not in others.

### Eukaryotes, operons, and noise

Improvement of dynamic performance of simple networks arising from cotranscription of interacting genes from the same operon raises the question of why operons are rare in eukaryotes. Eukaryotic cell volumes are much higher than prokaryotes, likely lowering the effect of intrinsic noise relative to the dominant effect of extrinsic noise [60]. Nevertheless, such benefits may still be present in some systems, and there are mechanisms that allow correlating gene expression noise in eukaryotic cells without polycistronic loci. Genes located near each other have correlated transcriptional bursts that likely arise from chromatin decondensation [61], [62]. Clusters of co-expressed genes, particularly metabolic genes, appear in eukaryotic chromosomes at a rate higher than would be expected randomly [63], [64]. Co-expressed, functionally related genes at distant genomic loci also appear to migrate together for co-transcription from discrete transcription initiation complexes [65], [66], [67]. These mechanisms, arising from the increased size and structural complexity of eukaryotic chromatin over prokaryotic chromosomes, can correlate gene expression noise with similar dynamic benefits to operons.

### Concluding remarks

We have developed a theory predicting that operon membership can increase or decrease noise in different types of protein interactions. Bioinformatic analysis finds that naturally occurring operon patterns in *E. coli* correlate with reduction of biochemical noise. Nevertheless, it would be interesting to explore operon coupling frequencies in bacterial stress response systems known to favor population-level heterogeneity, such as stress responses in *B. subtilis*; the amplification of noise by underlying ultrasensitive switches in non-redundant network modules may be a potential mechanism of population-level heterogeneity.

The existence of implicit ultrasensitive switches also underscores the idea that dramatic non-linearities are likely present in many simple protein interaction networks. Our results suggest that ultrasensitive switches are likely undetectable in the wild-type configurations of well-adapted systems as a result of selection against them, but may be present in conditions with lower selective pressure, or recent evolutionary events. These switches nevertheless have important implications for genome evolution. Their effects, and the mechanisms for avoiding them, may in turn shape larger biochemical networks by changing global noise properties, and will be an important factor in designing synthetic networks.

## Materials and Methods

Symbolic manipulations and data analysis were performed in Mathematica 7.0 and 8.0 (Wolfram Research, Champagne, IL). We predicted intrinsic noise characteristics with stochastic simulations at stationary state using the StochKit (http://engineering.ucsb.edu/~cse/StochKit/) tau-leaping routine for 10,000 runs of each model (except in the *lac* operon model, for which 1,000 runs of each condition were done). Initial model construction and test runs were done with Copasi (www.copasi.org). Simulations with an extrinsic noise representation were done in Copasi as detailed below. All models were represented with elementary reaction steps; in models involving gene regulation, we defined a promoter variable as always present at one copy per cell.

### Stochastic simulations

Unless otherwise specified, each network module was tested with promoter-mediated noise, represented by promoters switching between “on” and “off” states of mRNA production. This process has estimated switching rates of *k_goff_* = 0.0028 s^−1^ for switching to the “off” state and *k_gon_* = 0.00045 s^−1^ for switching to the “on” state [20]. In models including a gene regulation step, we assumed binding and unbinding of regulators to be independent of promoters switching between on and off states. Without promoter-generated bursting, gene expression noise largely arises from low mRNA copy numbers per cell and the effect of transcriptional coupling is qualitatively similar (Figure 1). Furthermore, analytical results from LNA do not include the effects of bursty transcription, showing that we arrive at qualitatively similar results without transcriptional bursts.

We distinguish between three types of coupling between production of two proteins in stochastic simulation reaction schemes. Transcription may be coupled or uncoupled (i.e., proteins in the same or separate operons) and when transcription is coupled, proteins may be cotranslated (single ribosome binding site for both) or translationally uncoupled (two ribosome binding sites). These three cases represent simplified extremes; intermediate translational linkage (e.g., read-through from multiple ribosome binding sites) is possible but was not further considered here. Figure 1 illustrates the three cases with promoter-mediated gene expression noise. For simplicity of presentation, we compare transcriptionally uncoupled with cotranslated models in the main text.

Tables S4, S5, S6, S7, S8 give reaction schematics and parameters for the gene expression and posttranslational models used in the main text. Parameter values were chosen to be of the correct order of magnitude for realistic expression levels and binding kinetics. To ensure a fair comparison between cotranscribed and uncoupled configurations, production and degradation rates of mRNA species for proteins A and B are identical. The degradation rate *k_deg_* corresponds to the value expected from a dilution rate for typical *E. coli* doubling every half hour.

### Analytical determination of protein covariance

The basis of noise differences between networks with proteins in the same operon and those with proteins in separate operons is the covariance between the expressed proteins. We used LNA to analytically characterize noise and covariance as follows. For the mean values of copy numbers (denoted by angular brackets):
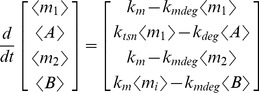
(8)where *i* = 1 with proteins *A* and *B* in the same operon, and *i* = 2 with proteins *A* and *B* in separate operons. Then we solved the fluctuation-dissipation matrix equation at steady state (**M**σ+σ**M** ^T^+Ω**N** = 0) for σ, where **M** is the Jacobian of the (macroscopic) system, Ω is cell volume, and **N** is the diffusion matrix [18]. Characterizing intrinsic noise as 
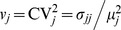
 and 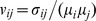 with indices *i* and *j* taking values corresponding to molecular species (*A, B, m*_1_ and m*_2_*), we follow the methods of [18] to obtain:
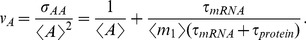
(9)and 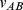 as in Equation 1. Note that *σ_AA_* is the variance, or the square of the standard deviation.

To analytically approximate noise of physiologically relevant variables in the simple network modules (Figure 2), we made the following simplifications to make the systems tractable. For metabolic steps with a substrate as a dependent variable, we assumed a Michaelis-Menten propensity. For the covalent modification module, we assumed a simple mass-action with no saturation or complexes. For the multiple gene regulator module, we used a Hill equation propensity for regulated mRNA production. Details of the analysis are in Text S2. Mean-field models are given in Table S3.

### Trends in *E. coli* K12 MG1655 genes

Bioinformatic analyses used pairs of interacting genes extracted from databases of *E. coli* K12 MG1655 as described below. To determine the randomized control, we needed to account for potential biases resulting from dataset size and other features of chromosome organization that we were not attempting to test. For instance, if we randomly assigned genes to extant operons in *E. coli* across the entire chromosome, the less frequently occurring modules would have much less same-operon membership than the modules with larger numbers of members, and would not be a useful control. We chose to randomize the genes extracted from the pairs within each module to set the random control for each class. Thus, for a list of gene pairs
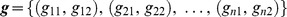
we determined a randomized case by flattening ***g*** into
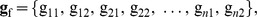
randomly permuting the order of the genes and then re-pairing them to determine the frequency. This process was repeated 1000 times to determine the parameters of the randomized distribution.

#### Metabolic networks

The *E. coli* metabolic network was extracted from Kyoto Encyclopedia of Genes and Genomes (KEGG; [29]). We partitioned the network into pairs of adjacent steps and assigned a value of 1 to each pair in the same operon (using the operon membership dataset from RegulonDB; [31]) and 0 to each pair in separate operons. This gave a frequency *f_met_* of subsequent same-operon metabolic steps. Several metabolic steps were found to be catalyzed by enzymes with subunits from multiple genes. Because such interactions have a large bias in favor of operon membership (Table 1), we eliminated them from the analysis to ensure operon membership biases result from metabolic, and not physical, interactions. We then repeatedly randomly assigned the metabolic enzymes to operons to generate a predicted distribution of background operon membership. The resulting distribution is approximately normal, allowing a parametric determination of *f_met_* significance (*f_met_* = 91/2417 = 0.038; mean μ_r_ = 0.0036; standard deviation σ_r_ = 0.0011; *p*<<10^−6^).

We extracted pairs of enzymes catalyzing the same metabolic step from the database to assess the trend in redundant metabolic steps. Here, *f*_redundant_ = 0/114. The resulting randomized distribution had mean μ_r_ = 0.0047; standard deviation σ_r_ = 0.0015 (*p*<<10^−6^ against this distribution). Enzymes catalyzing steps after a metabolic branch point were determined on the basis of common substrates in KEGG (*f*_branch_ = 5/2036; *p* = 0.071 against a randomized operon membership set of the same genes).

#### Covalent modifications

Extracting examples of this interaction type from databases is difficult because of the complex nature of the relationship, which requires interaction with subsequent modification. We used the MultiFun Gene Ontology from EcoCyc [30] to identify protein-related information transfer systems, each of which we manually checked to eliminate irrelevant cases and identify operon membership of the pair. Forty-six of the 201 pairs (23%) had interactions within the same operon. Because some of the identified members have multiple interaction pairs that could not be identified, the 46/201 fraction is somewhat uncertain. Unlike any of the other interaction modules, this module has pairs that overlap with another module (specifically, the physical interaction module). We used the same randomization procedure as for the metabolic pairs above to test the fraction relative to a randomized case of the covalent modification genes. With μ_c_ = 0.0047, σ_c_ = 0.0047, and *p*<<10^−6^, the true fraction is likely greater than the randomized control.

#### Multiple gene regulators

We used the *E. coli* gene regulation network from RegulonDB [31] to construct a graph of genes regulated by two or more regulators, excluding pairs that form subunits of a larger regulator. Following the method for metabolic networks, we assigned to each pair a value of 1 if the regulators are in the same operon and 0 if the regulators are in separate operons, giving frequency *f_g_* = 2/350 = 0.0057. Here, the random distribution has μ_rg_ = 0.099, σ_rg_ = 0.016, and *p*<<10^−6^.

#### Physical protein interactions

For physical protein interactions we used a functional interaction dataset [40], [41] to characterize the frequency of same-operon pairs, *f*_p_ = 0.35. In the randomized distribution, μ_p_ = 0.0030, σ_p_ = 0.00073, and *p*<<10^−6^.

### Operon relationship to protein copy number per cell

We extracted single-cell mRNA expression data (RNAseq) and protein copy number data from Taniguchi *et al* [43]. To ensure a meaningful comparison of expression levels, we considered only genes predicted to be unregulated in RegulonDB. Only the physical interaction module left enough data for analysis. For instance, the number of unregulated pairs in the same operon for the linear metabolic pathway dataset was 5, insufficient to distinguish the established operon membership pattern from noise when partitioned between high and low expression. Each average single-cell mRNA or protein copy number was used, along with physical interaction status (1 = same operon; 0 = non-same operon). Proteins with multiple interaction partners within and between operons were represented twice, once for same-operon and once for non-same-operon interaction. We then divided the set into above- and below-median subsets and compared the fraction of same-operon interactions in the subsets using a standard bootstrap resampling test. We resampled 10,000 times with replacement and computed the difference in coupling frequencies between low and high expression as the test statistic. To compute error bars, we used bootstrapping of each bin by sampling each bin with replacement up to the bin size, repeated 1,000 times.

## Supporting Information

Figure S1Conservation of noise relationships in network modules across expression level and parameter variations. Percentage noise difference is given by 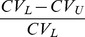; blue shades indicate lower CV for the coupled architecture; red, lower CV for the uncoupled architecture. A, J. Intermediate in linear metabolic pathway. Parameter: *k_cat2_*. The distributions at low expression level are highly skewed, giving misleading CV values that do not accurately reflect the higher variability in the uncoupled architecture. B, K. Product of metabolic pathway. Parameter: *k_cat2_*. C, L. Product of redundant metabolic step. Parameter: *k_cat1_* and *k_cat2_* varied simultaneously. D, M. Substrate of a metabolic branch point. Some cases have lower CV in the co-transcribed model because the distribution is bimodal, but the uncoupled model predicts lower actual variability than the co-transcribed model in all cases. Parameter: *k_cat1_* and *k_cat2_* varied simultaneously. E, N. Protein product of multiple gene regulator network. Parameter: *k_d_*. F, O. Monomer of physical protein interaction module. Parameter: *k_b_*. G, P. Heterodimer of physical protein interaction module. Parameter: *k_b_*. H, Q. Unmodified protein of covalent modification network. Parameter: *k_p_*. I, R. Modified protein of covalent modification network. Parameter: *k_p_* The scale for each variable was set by the largest absolute value. Expression levels (protein copy number/gene/cell) for multiple gene regulator module: L: 1 M: 53 H: 529. For all others: L: 53 M: 529 H: 5285.(PNG)

Figure S2 Non-redundant two-gene modules undergo an ultrasensitive switch dependent on production and degradation fluxes. Black lines are the mean-field steady state response while orange and blue lines trace timecourses from individual stochastic simulation trajectories. A. Intermediate in the linear metabolic pathway repeated from Figure 3C in the main text. B. Substrate levels at a metabolic branch point in response to changes in the balance between production and total consumption by two enzymes. Spikes are more likely when enzyme-mediated consumption fluxes at the branch point covary. C. Quantities of monomer subunit *A* of a heterodimer in response to different relative levels of *A* and *B* monomers. Physically interacting proteins produced asynchronously cross an ultrasensitive threshold, which is avoided by cotranscription from the same operon. D. Response of unmodified protein *A* in the covalent modification module to changes in the ratio of *A* to *B*. Unmodified protein *A* undergoes large spikes corresponding to crossing an ultrasensitive threshold when uncoupled.
(PNG)

Figure S3 Predicted dynamics of the *lac* operon system at three inducer concentrations. A. Simulated time courses of permease, allolactose and product. At all three concentrations, the transcriptionally uncoupled form of the system induces higher noise in allolactose (metabolic intermediate) concentration, but not product (glucose+galactose) or protein (permease). B. Correlations between permease and β-galactosidase in cotranscribed and uncoupled configurations. Throughout the range of induction the system demonstrates a consistent, significant reduction of correlation between permease and β-galactosidase in the uncoupled form of the system.
(PNG)

Figure S4 Effects of extrinsic noise on transcriptional coupling dynamics for metabolic modules. A. Linear metabolic pathway. B. Redundant metabolic step. Translational and transcriptional rate constants were randomly selected from uniform distributions to mimic global extrinsic noise. The resulting transcriptionally uncoupled protein distributions show a slight correlation between the proteins A and B (r = 0.293 top panel simulations and 0.388 in the bottom simulations). Metabolite noise differences between co-translated and transcriptionally uncoupled architectures are qualitatively unchanged from simulations that do not simulate extrinsic noise, with lower intermediate CV in the linear metabolic pathway (A) and higher product CV in the redundant metabolic step (B) in the cotranscribed configuration.
(PNG)

Figure S5 Distributions of linear metabolic and physically interacting protein pair interaction chromosomal locus distances in *E. coli* K12 MG1655. A. Gene pair locus distances in linear metabolic interactions are not distinguishable from randomized distances. B. A subset of gene pair locus distances in physical protein interactions have a distinct bias toward close chromosomal proximity. A distance randomization procedure (Text S4) does not indicate that proximity explains operon frequencies in either case.
(PNG)

Table S1 Predicted level of decorrelation between proteins that are uncoupled, cotranscribed, or cotranslated, with or without transcriptional bursting.
(PDF)

Table S2 Essential and nonessential subsets of gene pairs both have significantly high fractions of same-operon pairs *f*.
(PDF)

Table S3Mathematical models used for linear noise approximation of five simple network motifs in the form 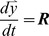.(PDF)

Table S4 Reactions for two genes expressed from the same and separate operons.
(PDF)

Table S5 Post-translational interactions in the linear and redundant metabolic step models.
(PDF)

Table S6 Post-translational interactions in the covalent modification and physical interaction models.
(PDF)

Table S7 Detailed *lac* operon model.
(PDF)

Table S8 Parameter values for detailed *lac* operon simulations.
(PDF)

Text S1 Decorrelation, extrinsic noise and expression levels.
(PDF)

Text S2 Analytical approach confirms predicted effects of cotranscription on intrinsic noise.
(PDF)

Text S3 Analytical determination of ultrasensitive thresholds.
(PDF)

Text S4 Chromosomal proximity of genes does not explain frequencies of metabolic or physical interaction operons.
(PDF)
