# Supplementary figures and images for: Interplay of Gene Expression Noise and Ultrasensitive Dynamics Affects Bacterial Operon Organization
Source: PLoS Comput Biol. 2012 Aug 30;8(8):e1002672. doi: 10.1371/journal.pcbi.1002672 (PMC3431296; doi:10.1371/journal.pcbi.1002672)

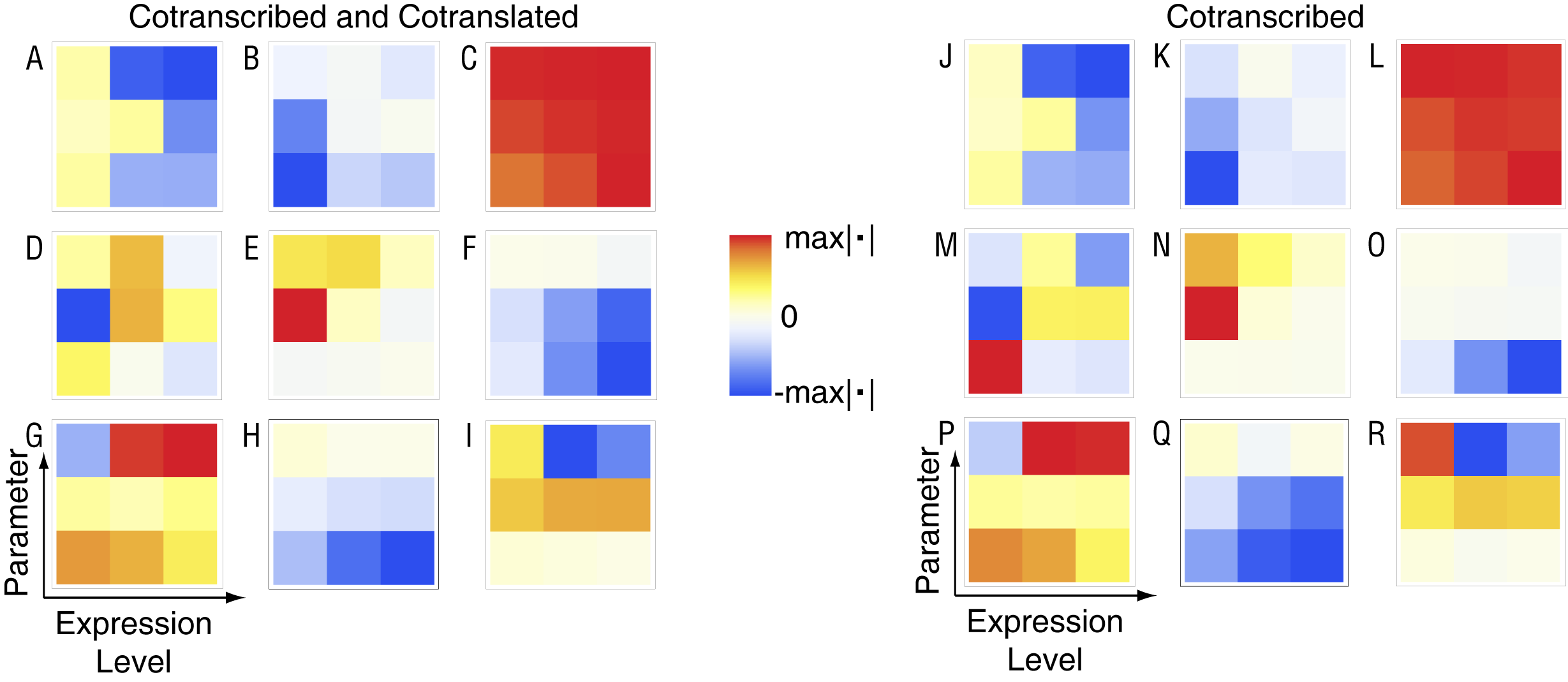

Supplement: Figure S1 — Conservation of noise relationships in network modules across expression level and parameter variations. Percentage noise difference is given by ; blue shades indicate lower CV for the coupled architecture; red, lower CV for the uncoupled architecture. A, J. Intermediate in linear metabolic pathway. Parameter: kcat2. The distributions at low expression level are highly skewed, giving misleading CV values that do not accurately reflect the higher variability in the uncoupled architecture. B, K. Product of metabolic pathway. Parameter: kcat2. C, L. Product of redundant metabolic step. Parameter: kcat1 and kcat2 varied simultaneously. D, M. Substrate of a metabolic branch point. Some cases have lower CV in the co-transcribed model because the distribution is bimodal, but the uncoupled model predicts lower actual variability than the co-transcribed model in all cases. Parameter: kcat1 and kcat2 varied simultaneously. E, N. Protein product of multiple gene regulator network. Parameter: kd. F, O. Monomer of physical protein interaction module. Parameter: kb. G, P. Heterodimer of physical protein interaction module. Parameter: kb. H, Q. Unmodified protein of covalent modification network. Parameter: kp. I, R. Modified protein of covalent modification network. Parameter: kp The scale for each variable was set by the largest absolute value. Expression levels (protein copy number/gene/cell) for multiple gene regulator module: L: 1 M: 53 H: 529. For all others: L: 53 M: 529 H: 5285. (PNG) [file pcbi.1002672.s001.png]

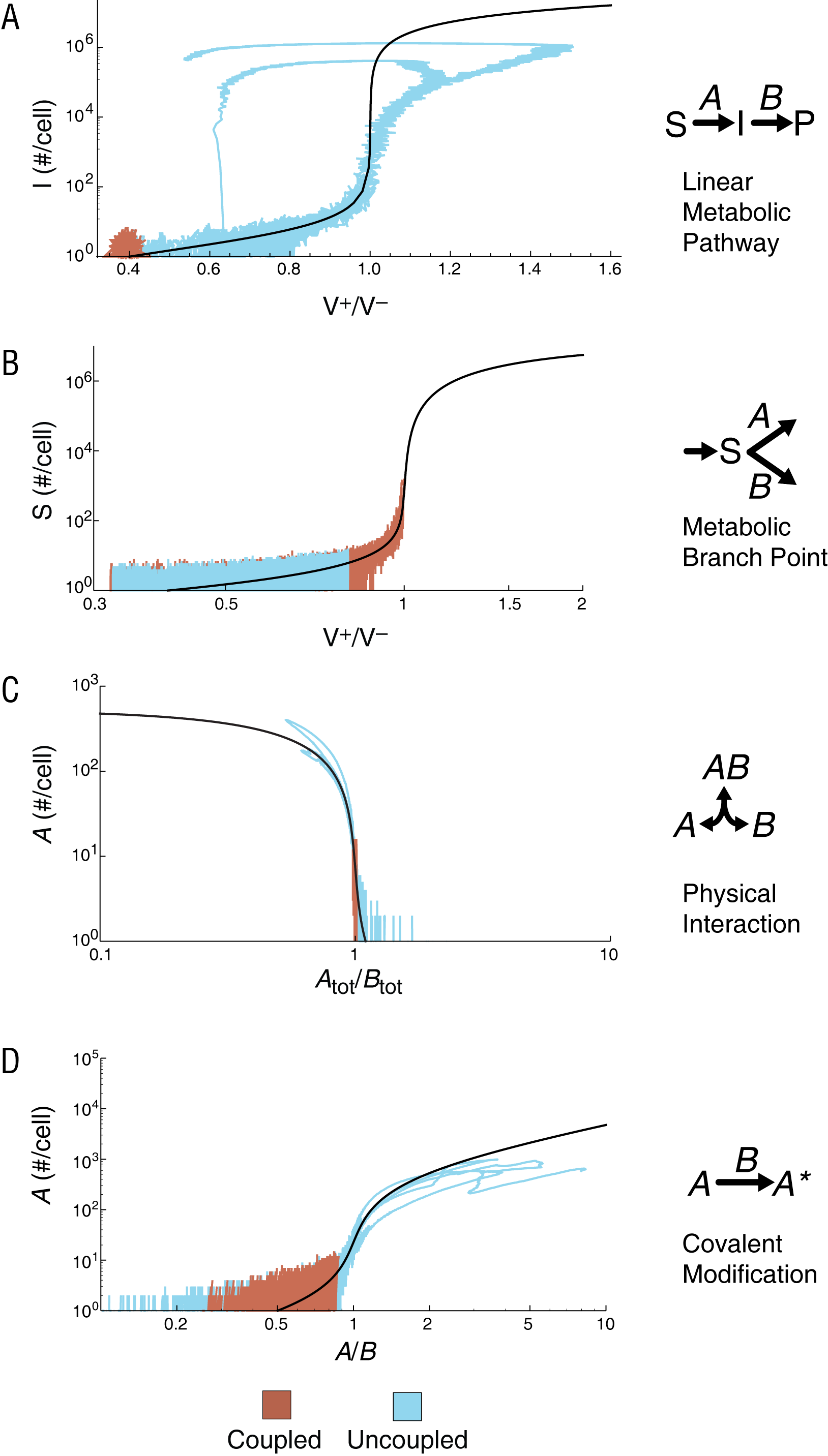

Supplement: Figure S2 — Non-redundant two-gene modules undergo an ultrasensitive switch dependent on production and degradation fluxes. Black lines are the mean-field steady state response while orange and blue lines trace timecourses from individual stochastic simulation trajectories. A. Intermediate in the linear metabolic pathway repeated from Figure 3C in the main text. B. Substrate levels at a metabolic branch point in response to changes in the balance between production and total consumption by two enzymes. Spikes are more likely when enzyme-mediated consumption fluxes at the branch point covary. C. Quantities of monomer subunit A of a heterodimer in response to different relative levels of A and B monomers. Physically interacting proteins produced asynchronously cross an ultrasensitive threshold, which is avoided by cotranscription from the same operon. D. Response of unmodified protein A in the covalent modification module to changes in the ratio of A to B. Unmodified protein A undergoes large spikes corresponding to crossing an ultrasensitive threshold when uncoupled. (PNG) [file pcbi.1002672.s002.png]

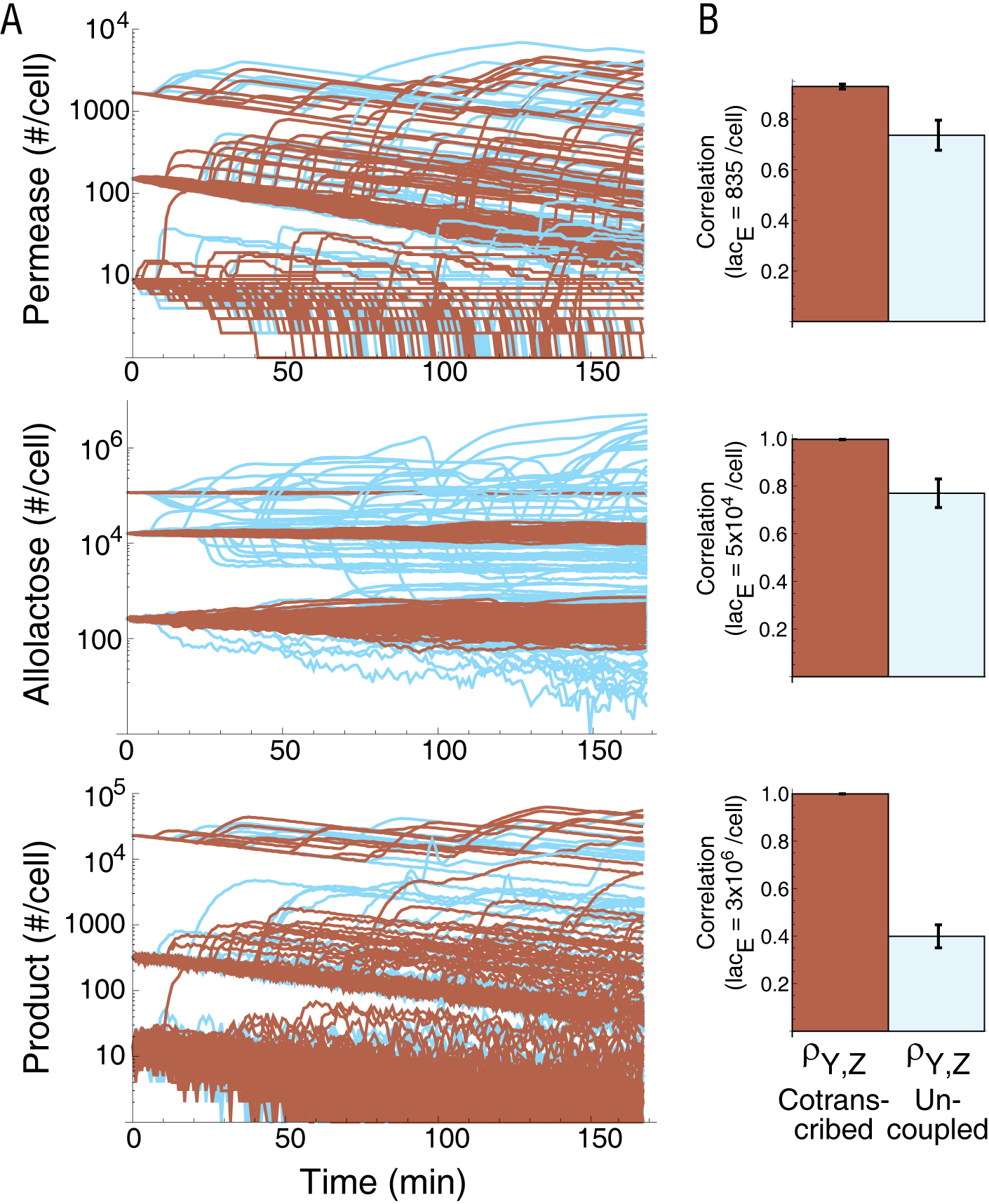

Supplement: Figure S3 — Predicted dynamics of the lac operon system at three inducer concentrations. A. Simulated time courses of permease, allolactose and product. At all three concentrations, the transcriptionally uncoupled form of the system induces higher noise in allolactose (metabolic intermediate) concentration, but not product (glucose+galactose) or protein (permease). B. Correlations between permease and β-galactosidase in cotranscribed and uncoupled configurations. Throughout the range of induction the system demonstrates a consistent, significant reduction of correlation between permease and β-galactosidase in the uncoupled form of the system. (PNG) [file pcbi.1002672.s003.png]

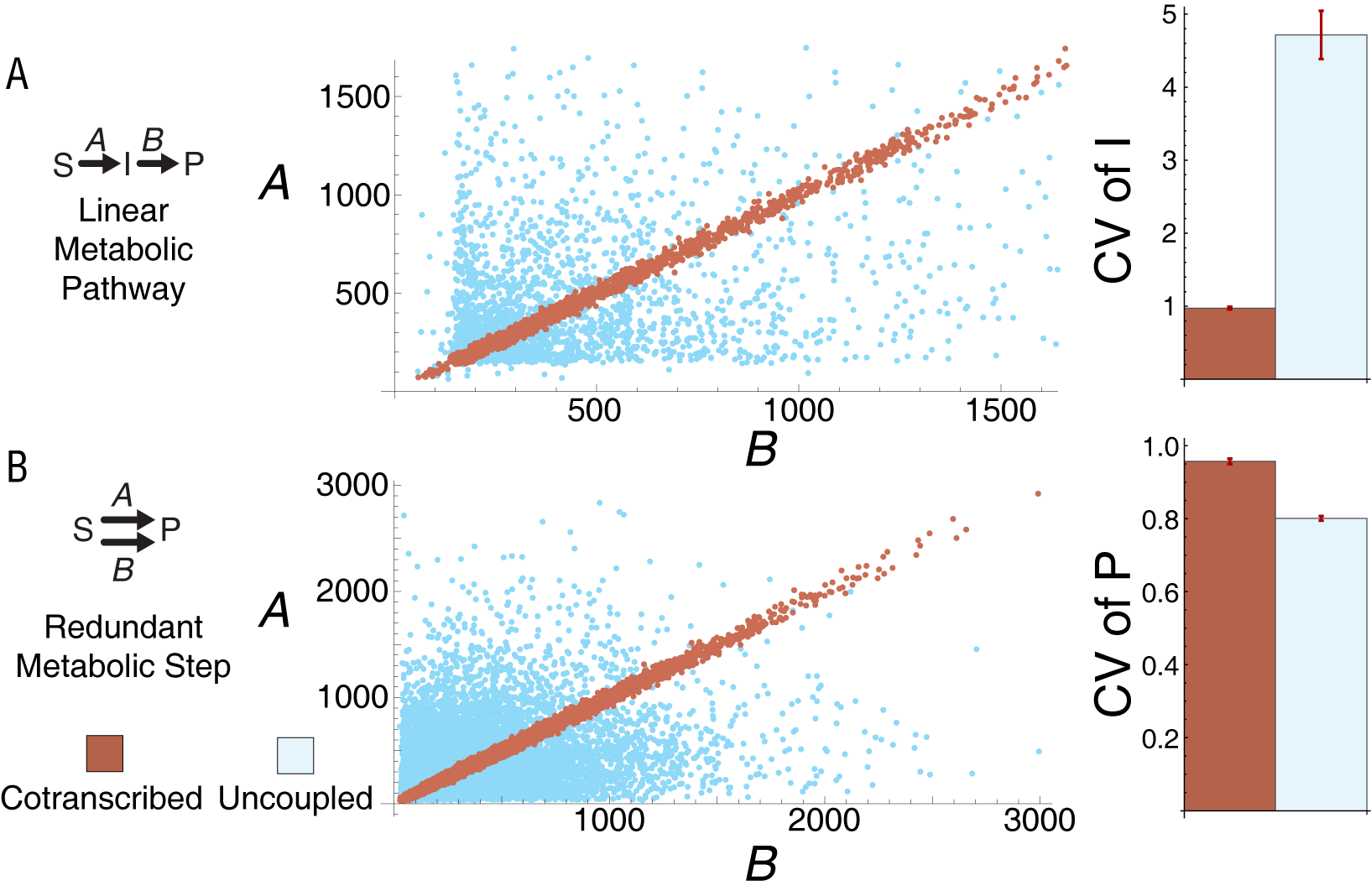

Supplement: Figure S4 — Effects of extrinsic noise on transcriptional coupling dynamics for metabolic modules. A. Linear metabolic pathway. B. Redundant metabolic step. Translational and transcriptional rate constants were randomly selected from uniform distributions to mimic global extrinsic noise. The resulting transcriptionally uncoupled protein distributions show a slight correlation between the proteins A and B (r = 0.293 top panel simulations and 0.388 in the bottom simulations). Metabolite noise differences between co-translated and transcriptionally uncoupled architectures are qualitatively unchanged from simulations that do not simulate extrinsic noise, with lower intermediate CV in the linear metabolic pathway (A) and higher product CV in the redundant metabolic step (B) in the cotranscribed configuration. (PNG) [file pcbi.1002672.s004.png]

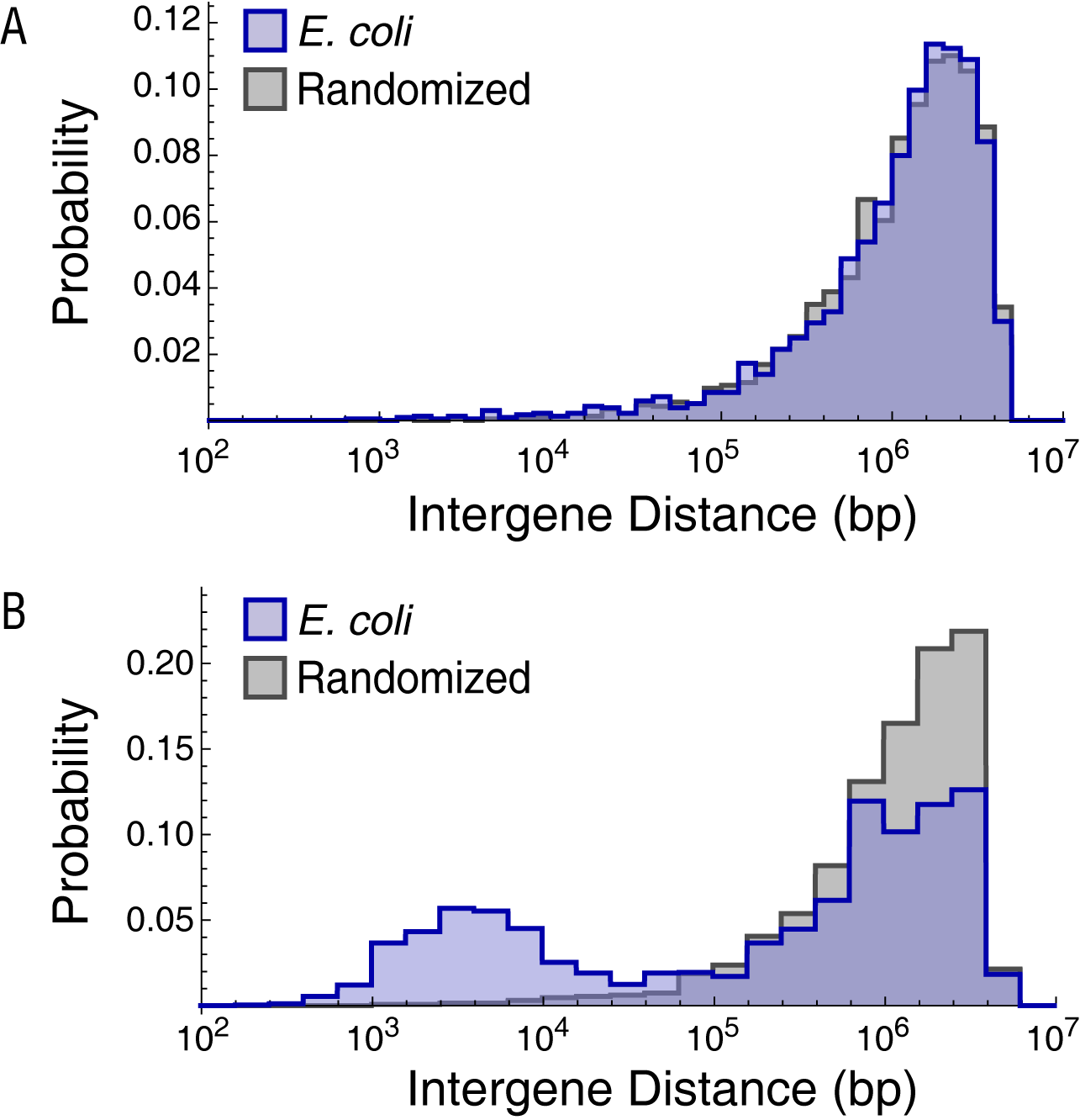

Supplement: Figure S5 — Distributions of linear metabolic and physically interacting protein pair interaction chromosomal locus distances in E. coli K12 MG1655. A. Gene pair locus distances in linear metabolic interactions are not distinguishable from randomized distances. B. A subset of gene pair locus distances in physical protein interactions have a distinct bias toward close chromosomal proximity. A distance randomization procedure (Text S4) does not indicate that proximity explains operon frequencies in either case. (PNG) [file pcbi.1002672.s005.png]
