# Supplementary material for: Interplay of Gene Expression Noise and Ultrasensitive Dynamics Affects Bacterial Operon Organization
Source: PLoS Comput Biol. 2012 Aug 30;8(8):e1002672. doi: 10.1371/journal.pcbi.1002672 (PMC3431296; doi:10.1371/journal.pcbi.1002672)
Supplement: Table S1 — Predicted level of decorrelation between proteins that are uncoupled, cotranscribed, or cotranslated, with or without transcriptional bursting. (PDF) [file pcbi.1002672.s006.pdf]

Table S1. Predicted level of decorrelation between proteins that are uncoupled, cotranscribed, or cotranslated, with or without transcriptional bursting

	Without transcriptional bursting			With transcriptional bursting		
Average expression level (#/cell)	Uncoupled	Cotranscribed	Cotranslated	Uncoupled	Cotranscribed	Cotranslated
52	0.29	0.019	0.0096	0.66	0.19	0.0094
534	0.026	0.0018	0.00091	0.38	0.0019	0.00094
5226	0.0028	0.00019	0.000092	0.35	0.00019	0.000092
