# Supplementary material for: Interplay of Gene Expression Noise and Ultrasensitive Dynamics Affects Bacterial Operon Organization
Source: PLoS Comput Biol. 2012 Aug 30;8(8):e1002672. doi: 10.1371/journal.pcbi.1002672 (PMC3431296; doi:10.1371/journal.pcbi.1002672)
Supplement: Table S2 — Essential and nonessential subsets of gene pairs both have significantly high fractions of same-operon pairs f. (PDF) [file pcbi.1002672.s007.pdf]

Table S2. Essential and nonessential subsets of gene pairs both have significantly high fractions of same-operon pairs *f*. The analysis was performed only for cases showing higher than random same-operon membership.

Network module	Number of essential cases	$f^{*}_{\text{essential}}$	$f^{rand}_{essential}$	Number of nonessential cases	$f^{*}_{\text{nonessential}}$	$f^{rand}_{\text{nonessential}}$
A. Linear metabolic pathway	139	0.029**	0.0094	399	0.033**	0.0065
B. Covalent modification	0	ND	ND	9	0.67	ND
C. Physical interaction	157	0.19**	0.0070	398	0.29**	0.0053

* Essential and nonessental gene subsets as designated by Taniguchi *et al* (2010).

** $p < 10^{-6}$ compared to f^{rand}.

ND: insufficient data for analysis.
