# Supplementary material for: Interplay of Gene Expression Noise and Ultrasensitive Dynamics Affects Bacterial Operon Organization
Source: PLoS Comput Biol. 2012 Aug 30;8(8):e1002672. doi: 10.1371/journal.pcbi.1002672 (PMC3431296; doi:10.1371/journal.pcbi.1002672)
Supplement: Table S3 — Mathematical models used for linear noise approximation of five simple network motifs in the form . (PDF) [file pcbi.1002672.s008.pdf]

Table S3. Mathematical models used for linear noise approximation of five simple network motifs in the form $\frac{d\vec{y}}{dt} = \boldsymbol{R}$. For species m_i, i = 1 in the coupled form and 2 in the uncoupled form.

Model	$\vec{y}^*$	$\boldsymbol{R}$
Gene expression	$\begin{bmatrix}\langle m_1\rangle\\ \langle A\rangle\\ \langle m_2\rangle\\ \langle B\rangle\end{bmatrix}$	$\begin{bmatrix}k_m - k_{mdeg}\langle m_1\rangle\\ k_{tsn}\langle m_1\rangle - k_{deg}\langle A\rangle\\ k_m - k_{mdeg}\langle m_2\rangle\\ k_{tsn}\langle m_i\rangle - k_{deg}\langle B\rangle\end{bmatrix}$
Linear metabolic pathway	$\begin{bmatrix}\langle A\rangle\\ \langle B\rangle\\ \langle I\rangle\\ \langle P\rangle\end{bmatrix}$	$\begin{bmatrix}k_{tsn}\langle m_1\rangle - k_{deg}\langle A\rangle\\ k_{tsn}\langle m_i\rangle - k_{deg}\langle B\rangle\\ v_1\langle A\rangle - v_2\frac{\langle I\rangle\langle B\rangle}{K_m+\langle I\rangle} - k_{deg}\langle I\rangle\\ v_2\frac{\langle I\rangle\langle B\rangle}{K_m+\langle I\rangle} - k_u\langle P\rangle\end{bmatrix}$
Redundant metabolic step	$\begin{bmatrix}\langle A\rangle\\ \langle B\rangle\\ \langle P\rangle\end{bmatrix}$	$\begin{bmatrix}k_{tsn}\langle m_1\rangle - k_{deg}\langle A\rangle\\ k_{tsn}\langle m_i\rangle - k_{deg}\langle B\rangle\\ v_1\langle A\rangle + v_2\langle B\rangle - k_u\langle P\rangle\end{bmatrix}$
Metabolic branch point	$\begin{bmatrix}\langle S\rangle\\ \langle A\rangle\\ \langle B\rangle\end{bmatrix}$	$\begin{bmatrix}k_{in} - k_1\frac{\langle S\rangle\langle A\rangle}{K_m+\langle A\rangle} - k_2\frac{\langle S\rangle\langle B\rangle}{K_m+\langle B\rangle} - k_{deg}\langle S\rangle\\ k_{tsn}\langle m_1\rangle - k_{deg}\langle A\rangle\\ k_{tsn}\langle m_i\rangle - k_{deg}\langle B\rangle\end{bmatrix}$
Covalent modification	$\begin{bmatrix}\langle A\rangle\\ \langle B\rangle\\ \langle AB\rangle\\ \langle A^*\rangle\end{bmatrix}$	$\begin{bmatrix}k_{tsn}\langle m_1\rangle - k_b\langle A\rangle\langle B\rangle - k_{deg}\langle A\rangle\\ k_{tsn}\langle m_i\rangle - k_b\langle A\rangle\langle B\rangle + k_p\langle AB\rangle - k_{deg}\langle B\rangle\\ k_b\langle A\rangle\langle B\rangle - k_p\langle AB\rangle - k_{deg}\langle AB\rangle\\ k_p\langle AB\rangle - k_{deg}\langle A^*\rangle\end{bmatrix}$
Multiple gene regulators	$\begin{bmatrix}\langle A\rangle\\ \langle B\rangle\\ \langle m_P\rangle\\ \langle P\rangle\end{bmatrix}$	$\begin{bmatrix}k_{tsn}\langle m_1\rangle - k_{deg}\langle A\rangle\\ k_{tsn}\langle m_i\rangle - k_{deg}\langle B\rangle\\ k_{mpb} + k_{mp}\frac{\langle A\rangle+\langle B\rangle}{K_m+\langle A\rangle+\langle B\rangle} - k_{mdeg}\langle m_P\rangle\\ k_{tsnp}\langle m_P\rangle - k_{deg}\langle P\rangle\end{bmatrix}$
Stoichiometric interaction	$\begin{bmatrix}\langle A\rangle\\ \langle B\rangle\\ \langle AB\rangle\end{bmatrix}$	$\begin{bmatrix}k_{tsn}\langle m_1\rangle - k_b\langle A\rangle\langle B\rangle + k_d\langle AB\rangle - k_{deg}\langle A\rangle\\ k_{tsn}\langle m_i\rangle - k_b\langle A\rangle\langle B\rangle + k_d\langle AB\rangle - k_{deg}\langle B\rangle\\ k_b\langle A\rangle\langle B\rangle - k_d\langle AB\rangle - k_{deg}\langle AB\rangle\end{bmatrix}$

*Variable definitions: m_1, m_2, m_p: mRNA. *A*, *B*: expressed proteins. *AB*: protein complex. A^*: Modified form of *A*. *S*, *I*: metabolic substrate and intermediates. *P*: metabolic product, or protein gene product in the Multiple Gene Regulator model.
