# Supplementary material for: Interplay of Gene Expression Noise and Ultrasensitive Dynamics Affects Bacterial Operon Organization
Source: PLoS Comput Biol. 2012 Aug 30;8(8):e1002672. doi: 10.1371/journal.pcbi.1002672 (PMC3431296; doi:10.1371/journal.pcbi.1002672)
Supplement: Table S4 — Reactions for two genes expressed from the same and separate operons. (PDF) [file pcbi.1002672.s009.pdf]

Table S4. Reactions for two genes expressed from the same and separate operons.

Reaction number	Reaction	Propensity	Parameter values
1	$\rightarrow mRNA_1$	$k_m g_1^*$	5×10^{-5}; 0.005; 0.05; 0.5**
2	$\rightarrow mRNA_2$	$k_m g_2^*$	5×10^{-5}; 0.005; 0.05; 0.5**
3	$mRNA_1 \rightarrow Ø$	$k_{mdeg} mRNA_1$	0.0033 s^{-1}
4†	$mRNA_i \rightarrow Ø$	$k_{mdeg} mRNA_i$	0.0033 s^{-1}
5	$\rightarrow A$	$k_{tsn} mRNA_1$	0.05 s^{-1}
6†	$\rightarrow B$	$k_{tsn} mRNA_i$	0.05 s^{-1}
6a‡	$\rightarrow A + B$	$k_{tsn} mRNA_1$	0.05 s^{-1}
7	$A \rightarrow Ø$	$k_{deg} A$	0.0002 s^{-1}
8	$B \rightarrow Ø$	$k_{deg} B$	0.0002 s^{-1}
9	$g_1 \rightleftharpoons g_1^*$	$k_{gon} g_1, k_{goff} g_1^*$	k_{gon} = 0.00045 s^{-1}
11	$g_2 \rightleftharpoons g_2^*$	$k_{gon} g_2, k_{goff} g_2^*$	k_{goff} = 0.0028 s^{-1}§

†In transcriptionally coupled models, $i = 1$; in uncoupled models, $i = 2$.

‡In translationally coupled models, use reaction 6a in place of reactions 5 and 6.

** Parameter k_m assumes slow promoter kinetics. Multiply values by 0.1395 to get equivalent expression for fast promoter kinetics ($k_{goff} = 0$). Four values represent the scan shown in Figure S1. The lower three used for multiple gene regulator module; the higher three used for all others. Expression levels used for examples in Figure 2: linear metabolic, 0.5; redundant metabolic, 0.5; divergent metabolic, 0.5; covalent modification, 0.05; multiple gene regulators, 5×10^{-5}; physical interaction, 0.05.

§$k_{goff} = 0$ for fast promoter kinetics.
