# Supplementary material for: Interplay of Gene Expression Noise and Ultrasensitive Dynamics Affects Bacterial Operon Organization
Source: PLoS Comput Biol. 2012 Aug 30;8(8):e1002672. doi: 10.1371/journal.pcbi.1002672 (PMC3431296; doi:10.1371/journal.pcbi.1002672)
Supplement: Table S5 — Post-translational interactions in the linear and redundant metabolic step models. (PDF) [file pcbi.1002672.s010.pdf]

Table S5. Post-translational interactions in the linear and redundant metabolic step models. These reactions are combined with those from Table S4 to create uncoupled, transcriptionally coupled, and translationally coupled model forms.

Linear metabolic pathway			
Reaction number	Reaction*	Propensity**	Parameter values
1	$A + S \rightleftharpoons A.S$	$k_1 A,\ k_{-1} A.S$	2 s^{-1}†, 2 s^{-1}
2	$A.S \rightarrow A + I$	$k_{cat1} A.S$	1 s^{-1}
3	$B + I \rightleftharpoons B.I$	$k_2 B \cdot I, k_{-2} B.I$	2 Ω/(#×s), 2 s^{-1}
4	$B.I \rightarrow B + P$	$k_{cat2} B.I$	0.5; 1; 2 s^{-1}‡
5	$A.S \rightarrow \text{Ø}$	$k_{deg} A.S$	0.0002 s^{-1}
6	$B.I \rightarrow \text{Ø}$	$k_{deg} B.I$	0.0002 s^{-1}
7	$I \rightarrow \text{Ø}$	$k_{deg} I$	0.0002 s^{-1}
8	$P \rightarrow \text{Ø}$	$k_u P$	2 s^{-1}
**Redundant metabolic step**			
Reaction number	Reaction*	Propensity**	Parameter values
1	$A + S \rightleftharpoons A.S$	$k_1 A \cdot S,\ k_{-1} A.S$	2 s^{-1}†, 2 s^{-1}
2	$A.S \rightarrow A + P$	$k_{cat1} A.S$	0.5; 1; 2 s^{-1}‡
3	$B + S \rightleftharpoons B.S$	$k_2 B \cdot S,\ k_{-2} B.S$	2 s^{-1}†, 2 s^{-1}
4	$B.S \rightarrow B + P$	$k_{cat2} B.S$	0.5; 1; 2 s^{-1}‡
5	$A.S \rightarrow \text{Ø}$	$k_{deg} A.S$	0.0002 s^{-1}
6	$B.S \rightarrow \text{Ø}$	$k_{deg} B.S$	0.0002 s^{-1}
7	$P \rightarrow \text{Ø}$	$k_u P$	2 s^{-1}
**Metabolic branch point**			
Reaction number	Reaction*	Propensity	Parameter values
1	$\rightarrow S$	k_{in}	4228.98 s^{-1}
1	$A + S \rightleftharpoons A.S$	$k_1 A \cdot S,\ k_{-1} A.S$	2 s^{-1}†, 2 s^{-1}
2	$A.S \rightarrow A$	k_{cat1}A.S	0.5; 1; 6 s^{-1}‡
3	$B + S \rightleftharpoons B.S$	$k_2 B \cdot S,\ k_{-2} B.S$	2 s^{-1}†, 2 s^{-1}
4	$B.S \rightarrow B$	$k_{cat2} B.S$	0.5; 1; 6 s^{-1}‡
5	$A.S \rightarrow \text{Ø}$	$k_{deg} A.S$	0.0002 s^{-1}
6	$B.S \rightarrow \text{Ø}$	$k_{deg} B.S$	0.0002 s^{-1}

*Variable definitions: *A*, *B*: metabolic enzymes; *S:* substrate; *I:* intermediate; *P:* product.

**Unbound substrate *S* is held constant and subsumed into parameter k_1.

†k_1 (and k_2 in the redundant metabolic step only) is a composite parameter equal to [precursor]×V_i where units of V_i are Ω/(#×s); Ω represents cell volume.

‡Multiple entries denote scan values for Figure S1. In the redundant step and branch point, k_{cat1} and k_{cat2} were varied simultaneously. Main text example used 1 for linear redundant pathway and 6 for metabolic branch point.
