# Supplementary material for: Interplay of Gene Expression Noise and Ultrasensitive Dynamics Affects Bacterial Operon Organization
Source: PLoS Comput Biol. 2012 Aug 30;8(8):e1002672. doi: 10.1371/journal.pcbi.1002672 (PMC3431296; doi:10.1371/journal.pcbi.1002672)
Supplement: Table S6 — Post-translational interactions in the covalent modification and physical interaction models. (PDF) [file pcbi.1002672.s011.pdf]

Table S6. Post-translational interactions in the covalent modification and physical interaction models. These reactions are combined with those from Table S4 to create uncoupled, transcriptionally coupled, and translationally coupled model forms. $g^?$ denotes a promoter wildcard that can be on (g^*) or off (g).

Covalent modification model			
Reaction number	Reaction	Propensity	Parameter values
1	$A + B \rightleftharpoons A.B$	$k_b A \cdot B,\ k_d A.B$	0.5 Ω/(#×s), 0.5 s^{-1}
2	$A.B \rightarrow A^* + B$	$k_p A.B$	10^{-8}; 0.0001; 1 s^{-1}†
3	$A.B \rightarrow Ø$	$k_{deg} A.B$	0.0002 s^{-1}
4	$A^* \rightarrow Ø$	$k_{deg} A^*$	0.0002 s^{-1}
**Physical interaction model**			
Reaction number	Reaction	Propensity	Parameter values
1	$A + B \rightleftharpoons A.B$	$k_b A \cdot B,\ k_d A.B$	0.0001; 0.038; 10 Ω/(#×s)†, 1 s^{-1}
2	$A.B \rightarrow Ø$	$k_{deg} A.B$	0.0002 s^{-1}
**Multiple gene regulator model**			
Reaction number	Reaction	Propensity	Parameter values
1	$\rightarrow mRNA_p$	$k_{mp} g_p^* .A$	0.05 s^{-1}
2	$\rightarrow mRNA_p$	$k_{mp} g_p^* .B$	0.05 s^{-1}
3	$\rightarrow mRNA_p$	$k_{mp} g_p^* .A.B$	0.05 s^{-1}
4	$mRNA_p \rightarrow Ø$	$k_{mdeg} mRNA_p$	0.05 s^{-1}
5	$\rightarrow P$	$k_{tsn} mRNA_p$	0.05 s^{-1}
6	$P \rightarrow Ø$	$k_{deg} P$	0.05 s^{-1}
7	$g_p \rightleftharpoons g_p^*$	$k_{gon} g_p, k_{goff} g_p^*$	0.00045, 0.0028 s^{-1}‡
8	$g_p^? + A \rightleftharpoons g_p^? A$	$k_b g_p^? \cdot A, k_d g_p^? A$	k_b = 1 Ω/(#×s), k_d = 1; 55; 100 s^{-1}†
9	$g_p^? + B \rightleftharpoons g_p^? B$	$k_b g_p^? \cdot B, k_d g_p^? B$	
10	$g_p^? A + B \rightleftharpoons g_p^? A.B$	$k_b g_p^? A \cdot B, k_d g_p^? A.B$	
11	$g_p^? B + A \rightleftharpoons g_p^? A.B$	$k_b g_p^? B \cdot A, k_d g_p^? A.B$	
12	$g_p A \rightleftharpoons g_p^* A$	$k_{gon} g_p A, k_{goff} g_p^* A$	0.00045, 0.0028 s^{-1}‡
13	$g_p B \rightleftharpoons g_p^* B$	$k_{gon} g_p B, k_{goff} g_p^* B$	0.00045, 0.0028 s^{-1}‡
14	$g_p A.B \rightleftharpoons g_p^* A.B$	$k_{gon} g_p A.B, k_{goff} g_p^* A.B$	0.00045, 0.0028 s^{-1}‡

†Multiple entries denote scan values for Figure S1. Main text example used 0.0001 for the covalent modification model, 10 for the physical interaction model, and 55 for the multiple gene regulator model.
‡k_{goff} = 0 for fast promoter kinetics.
