# Supplementary material for: Interplay of Gene Expression Noise and Ultrasensitive Dynamics Affects Bacterial Operon Organization
Source: PLoS Comput Biol. 2012 Aug 30;8(8):e1002672. doi: 10.1371/journal.pcbi.1002672 (PMC3431296; doi:10.1371/journal.pcbi.1002672)
Supplement: Table S7 — Detailed lac operon model. (PDF) [file pcbi.1002672.s012.pdf]

Table S7. Detailed *lac* operon model. $g^?$ denotes a promoter wildcard that can be on (g^*) or off (g).

Reaction number	Reaction	Propensity
1	$\rightarrow \mathrm{mRNA}_{lacY}\ (g^*_{lacY})$	$k_m g^*_{lacY}$
2	$\rightarrow \mathrm{mRNA}_{lacZ}\ (g^*_{lacZ})$	$k_m g^*_{lacZ}$
3	$\rightarrow \mathrm{mRNA}_{lacI}\ (g^*_{lacI})$	$k_{mI} g^*_{lacI}$
4	$\mathrm{mRNA} \rightarrow \emptyset$	$k_{mdeg}\mathrm{mRNA}$
5	$\rightarrow \mathrm{LacY}\ (\mathrm{mRNA}_{lacY})$	$k_{tsn}\mathrm{mRNA}_{lacY}$
6	$\rightarrow \mathrm{LacZ}\ (\mathrm{mRNA}_{lacZ})$	$k_{tsn}\mathrm{mRNA}_{lacZ}$
7	$\rightarrow \mathrm{LacI}\ (\mathrm{mRNA}_{lacI})$	$k_{tsn}\mathrm{mRNA}_{lacI}$
8	$\mathrm{LacY, Z, I, I_2, I_4} \rightarrow \emptyset$	$k_{deg}\mathrm{LacY, Z, I, I_2, I_4}$
9	$2\ \mathrm{LacI} \rightarrow \mathrm{LacI_2}$	$k_{lacI}\mathrm{LacI}\ (\mathrm{LacI}-1)$
10	$2\ \mathrm{LacI_2} \rightarrow \mathrm{LacI_4}$	$k_{lacI}\mathrm{LacI_2}\ (\mathrm{LacI_2}-1)$
11	$\mathrm{LacY} \rightleftharpoons \mathrm{LacY.lac_e}$	$k_{bl}\mathrm{LacY}, k_{dl}\mathrm{LacY.lac_e}$
12	$\mathrm{LacY.lac_e} \rightarrow \mathrm{LacY + lac}$	$k_{perm}\mathrm{LacY.lac_e}$
13	$\mathrm{LacZ + lac} \rightleftharpoons \mathrm{LacZ.lac}$	$k_{bp}\mathrm{LacZ}, k_{dp}\mathrm{LacZ.lac}$
14	$\mathrm{LacZ.lac} \rightleftharpoons \mathrm{LacZ + alac}$	$k_{dp2}\mathrm{LacZ.lac}, k_{bp2}\mathrm{LacZ + alac}$
15	$\mathrm{LacZ.lac} \rightarrow \mathrm{LacZ + p}$	$k_{cat}\mathrm{LacZ.lac}$
16	$\mathrm{LacI_4} + 2\ \mathrm{alac} \rightleftharpoons \mathrm{LacI_4.alac_2}$	$k_{ba}\mathrm{LacI_4} \cdot \mathrm{alac} \cdot (\mathrm{alac}-1),\ k_{da}\mathrm{LacI_4.alac_2}$
17	$\mathrm{LacI_4.alac_2} + 2\ \mathrm{alac} \rightleftharpoons \mathrm{LacI_4.alac_4}$	$k_{ba}\mathrm{LacI_4} \cdot \mathrm{alac_2} \cdot (\mathrm{alac_2}-1),\ k_{da}\mathrm{LacI_4.alac_4}$
18	$\mathrm{LacY.lac_e} \rightarrow \emptyset$	$k_{deg}\ \mathrm{LacY.lac_e}$
19	$\mathrm{LacZ.lac} \rightarrow \emptyset$	$k_{deg}\ \mathrm{LacZ.lac}$
20	$\mathrm{LacI_4.alac_2} \rightarrow \emptyset$	$k_{deg}\ \mathrm{LacI_4.alac_2}$
21	$\mathrm{LacI_4.alac_4} \rightarrow \emptyset$	$k_{deg}\ \mathrm{LacI_4.alac_4}$
22	$g_{lacY} \rightleftharpoons g^*_{lacY}$	$k_{gon} g_{lacY}, k_{goff} g^*_{lacY}$
23	$g_{lacZ} \rightleftharpoons g^*_{lacZ}$	$k_{gon} g_{lacZ}, k_{goff} g^*_{lacZ}$
24	$g_{lacI} \rightleftharpoons g^*_{lacI}$	$k_{gon} g_{lacI}, k_{goff} g^*_{lacI}$
25	$g^?_{lacY} + \mathrm{LacI_4} \rightleftharpoons g^?_{lacY}.\mathrm{LacI_4}$	$k_b g^?_{lacY} \cdot \mathrm{LacI_4}, k_d g^?_{lacY}\mathrm{LacI_4}$
26	$g^?_{lacZ} + \mathrm{LacI_4} \rightleftharpoons g^?_{lacZ}.\mathrm{LacI_4}$	$k_b g^?_{lacZ} \cdot \mathrm{LacI_4}, k_d g^?_{lacZ}\mathrm{LacI_4}$
27	$g^?_{lacY} + \mathrm{LacI_4.alac_2} \rightleftharpoons g^?_{lacY}.\mathrm{LacI_4.alac_2}$	$k_{b2} g^?_{lacY} \cdot \mathrm{LacI_4.alac_2}, k_{d2} g^?_{lacY}\mathrm{LacI_4.alac_2}$
28	$g^?_{lacZ} + \mathrm{LacI_4.alac_2} \rightleftharpoons g^?_{lacZ}.\mathrm{LacI_4.alac_2}$	$k_{b2} g^?_{lacZ} \cdot \mathrm{LacI_4.alac_2}, k_{d2} g^?_{lacZ}\mathrm{LacI_4.alac_2}$
29	$g_{lacY}.\mathrm{LacI_4} \rightleftharpoons g^*_{lacY}.\mathrm{LacI_4}$	$k_{gon} g_{lacY}.\mathrm{LacI_4}, k_{goff} g^*_{lacY}.\mathrm{LacI_4}$
30	$g_{lacZ}.\mathrm{LacI_4} \rightleftharpoons g^*_{lacZ}.\mathrm{LacI_4}$	$k_{gon} g_{lacZ}.\mathrm{LacI_4}, k_{goff} g^*_{lacZ}.\mathrm{LacI_4}$
31	$g_{lacY}.\mathrm{LacI_4.alac_2} \rightleftharpoons g^*_{lacY}.\mathrm{LacI_4.alac_2}$	$k_{gon} g_{lacY}.\mathrm{LacI_4.alac_2}, k_{goff} g^*_{lacY}.\mathrm{LacI_4.alac_2}$
32	$g_{lacZ}.\mathrm{LacI_4.alac_2} \rightleftharpoons g^*_{lacZ}.\mathrm{LacI_4.alac_2}$	$k_{gon} g_{lacZ}.\mathrm{LacI_4.alac_2}, k_{goff} g^*_{lacZ}.\mathrm{LacI_4.alac_2}$
33	$\mathrm{p} \rightarrow \emptyset$	$k_u\, p$
