# Supplementary material for: Interplay of Gene Expression Noise and Ultrasensitive Dynamics Affects Bacterial Operon Organization
Source: PLoS Comput Biol. 2012 Aug 30;8(8):e1002672. doi: 10.1371/journal.pcbi.1002672 (PMC3431296; doi:10.1371/journal.pcbi.1002672)
Supplement: Table S8 — Parameter values for detailed lac operon simulations. (PDF) [file pcbi.1002672.s013.pdf]

Table S8. Parameter values for detailed *lac* operon simulations.

Parameter	Interpretation	Value	Units
k_m	Transcription	0.5808	s^{-1}
k_{mI}	LacI Transcription	0.000167	s^{-1}
k_{mdeg}	mRNA degradation	0.00791	s^{-1}
k_{tsnY}	Translation of LacY	0.0833	s^{-1}
k_{tsnZ}	Translation of LacZ	0.0500	s^{-1}
k_{deg}	Protein dilution	0.0002	s^{-1}
k_{lacI}	LacI dimerization	0.167	Ω/(#×s)
k_{bl}	LacY-lac_e binding	5.00×10^{-05}	Ω/(#×s)
k_{dl}	LacY-lac_e unbinding	5.00	s^{-1}
k_{perm}	lac_e import	10.0	s^{-1}
k_{bp}	LacZ-lac binding	0.000563	Ω/(#×s)
k_{dp}	LacZ-lac unbinding	383	s^{-1}
k_{dp2}	lac conversion to alac	1670	s^{-1}
k_{bp2}	alac conversion to lac	0.00119	Ω/(#×s)
k_{cat}	lac conversion to product	253	s^{-1}
k_u	product use and dilution	1.67	s^{-1}
k_{ba}	alac-$LacI_4$ binding	1.67×10^{-06}	$\Omega^2/(\#^2\times s)$
k_{da}	alac-$LacI_4$ unbinding	167	s^{-1}
k_{gon}	Transcription on	0.000450	s^{-1}
k_{goff}	Transcription off	0.00278	s^{-1}
k_b	$LacI_4$-promoter binding	0.167	Ω/(#×s)
k_d	$LacI_4$-promoter unbinding	0.000244	s^{-1}
k_{b2}	$LacI_4.alac_2$-promoter binding	0.0167	Ω/(#×s)
k_{d2}	$LacI_4.alac_2$-promoter unbinding	0.00167	s^{-1}
k_u	Product utilization	1.67	s^{-1}
$lac_e(0)$	Extracellular lactose (lac_e)	835	#
