# Supplementary material for: Interplay of Gene Expression Noise and Ultrasensitive Dynamics Affects Bacterial Operon Organization
Source: PLoS Comput Biol. 2012 Aug 30;8(8):e1002672. doi: 10.1371/journal.pcbi.1002672 (PMC3431296; doi:10.1371/journal.pcbi.1002672)
Supplement: Text S1 — Decorrelation, extrinsic noise and expression levels. (PDF) [file pcbi.1002672.s014.pdf]

Text S1. Decorrelation, extrinsic noise and expression levels

Decorrelation as an inverse measure of the correlative effects of operons

The effect of operons on correlations between protein numbers in experimental data or stochastic simulations can be found by calculating the *degree of decorrelation* between the two protein copy numbers:

$$\delta_{AB} = \frac{1}{2}\left\langle \left(\frac{A}{\langle A \rangle} - \frac{B}{\langle B \rangle} \right)^2 \right\rangle . \quad \text{(S1.1)}$$

This quantity demonstrates how far the proteins fluctuate from being proportional to one another in a manner similar to a previously introduced intrinsic noise formula [67], and is be useful to assess the effects of gene expression level on noise differences between cotranscribed and uncoupled proteins. Table S1 shows δ_{AB} for expression of proteins that are uncoupled, cotranscribed, and cotranslated with or without transcriptional bursting.

As Table S1 shows, the most important intrinsic effect correlating proteins is operon cotranscription. However, δ_{AB} also shows that low gene expression levels have higher decorrelation in the uncoupled configuration, as compared to the cotranscribed case. Expression from an operon can also be translationally coupled, with a single ribosome binding site for the operon, as opposed to separate binding sites for each gene. Figure 1 and Table S1 indicate that translational coupling has only a minor effect on the level of decorrelation as compared with transcriptional coupling.

Extrinsic noise does not eliminate the effect of coupling at high expression levels

Global extrinsic noise is an important factor in cell-to-cell variation of protein copy number with two potentially important considerations. First, extrinsic noise exceeds intrinsic

noise in proteins with average single-cell copy numbers higher than approximately 10 per cell on average [42]. Second, extrinsic noise can cause expression levels of proteins in different operons to be correlated [42,67]. Therefore, the effects of operons on intrinsic noise need to be evaluated in the context of the extrinsic noise as well.

Notably, the covariance of cotranscribed proteins is predicted to be much larger than that of transcriptionally uncoupled proteins subject to extrinsic noise. For instance, Taniguchi *et al* [42] reported correlations between two proteins on the order of 0.6 arising from extrinsic noise, whereas the predicted correlations for cotranscribed proteins arising from intrinsic noise exceed 0.9 (Figure 1).

To further assess the effects of extrinsic noise, we ran stochastic simulations for the linear and redundant metabolic pathway network modules with extrinsic parameter variations (Figure S4). Our approach was to sample the rate constants for transcription and translation from uniform distributions (i.e., k_m, $k_{tsn} \in [0.01, 0.09]$). This simulates extrinsic correlations arising from RNA polymerase and ribosome copy numbers, which would be the same within a single bacterial cell but differ between cells. The remaining parameters were the same as those used in the examples in Figure 2 (Tables S4-S6). We ran the simulations (n = 10,000) using the "parameter scan" feature in Copasi to sample the parameters, running each parameter set a single time for 10,000 seconds of simulated time and taking only the final time point for analysis. Because extrinsic variations in RNA polymerase and ribosome copy numbers are expected to arise from binomial partitioning during cell division, our parameter sampling approach is statistically valid only for simulations at stationary state where we consider a single time point longer than the autocorrelation time of the system, as we use it here.

The results (Figure S4) confirm that the effects of cotranscription on intrinsic covariance remain evident at high gene expression levels (where extrinsic noise is greater than intrinsic noise). Enzyme cotranscription results in less variability in metabolic intermediate in the linear pathway and more variability in metabolic product in the redundant pathway, consistent with simulations lacking extrinsic noise (Figure 2).
