# Supplementary material for: Interplay of Gene Expression Noise and Ultrasensitive Dynamics Affects Bacterial Operon Organization
Source: PLoS Comput Biol. 2012 Aug 30;8(8):e1002672. doi: 10.1371/journal.pcbi.1002672 (PMC3431296; doi:10.1371/journal.pcbi.1002672)
Supplement: Text S2 — Analytical approach confirms predicted effects of cotranscription on intrinsic noise. (PDF) [file pcbi.1002672.s015.pdf]

Text S2. Analytical approach confirms predicted effects of cotranscription on intrinsic noise

To determine how generally the simulation results hold in the face of different parameter values, we used the linear noise approximation (LNA) to analytically determine noise differences between cotranscribed and uncoupled forms of each network module (here, the square coefficient of variation: $\nu = CV^2 = \sigma^2/\mu^2$). For each molecular species denoted by index j taking values that correspond the species names, we present the noise difference between cotranscribed (ν_j^C) and uncoupled (ν_j^U) configurations as $\Delta\nu_j = \nu_j^U - \nu_j^C$. If the value is positive, the cotranscribed configuration has lower CV^2; if it is negative, the uncoupled configuration has lower CV^2. Following Paulsson [18], the $\Delta\nu_j$ are characterized in terms of logarithmic gains of $V_j^+ \big/ V_j^-$ (the ratio of fluxes producing and consuming species j) with respect to concentration y_i:

$$H_{ij} = \frac{\partial \log(V_j^+ \big/ V_j^-)}{\partial \log y_i}.$$

Linear metabolic pathway

For the linear metabolic pathway, we have

$$\Delta\nu_I = \nu_{AB} \frac{2H_{AI}H_{BI}}{H_{II}(1-H_{II})} \frac{\tau_{mRNA} + \tau_{protein} - H_{II}\tau_{mRNA}}{\tau_{protein} - H_{II}\tau_{mRNA}}. \qquad \text{(S2.1)}$$

Using the well-known fact that mRNA has a shorter half-life than protein (i. e, $\tau_{mRNA} << \tau_{protein}$), $\Delta\nu_I$ simplifies to the following expression

$$\Delta\nu_I \approx \nu_{AB} \frac{2H_{AI}H_{BI}}{H_{II}(1-H_{II})}. \qquad \text{(S2.2)}$$

With the steady state flux through the first step in the pathway as φ_1 and the second step as φ_2, we have $H_{AI} = \frac{\varphi_1}{\varphi_1 - \varphi_2}$ and $H_{BI} = \frac{\varphi_2}{\varphi_2 - \varphi_1}$. At steady state, $\varphi_1 = \varphi_2 + \langle I \rangle / \tau_{protein}$ so that H_{AI} is positive

(increase in this enzyme increases production of the intermediate) and H_{BI} is negative (increase in this enzyme increases consumption of the intermediate). Gain H_{II} is given by $H_{II} = -\frac{\langle I \rangle}{K_m + \langle I \rangle} - \frac{K_m}{K_m + \langle I \rangle} H_{AI} = \frac{K_m}{K_m + \langle I \rangle} H_{BI} - 1$, where K_m is the Michaelis-Menten constant of enzyme B. Then $H_{II} < -1$; in particular, $H_{II} \approx -1$ if enzyme B is operating close to saturation (which is typical; [45]). Then the denominator $H_{II}(1 - H_{II})$ is negative and we have a lower bound:

$$\Delta v_I > v_{AB}. \tag{S2.3}$$

Thus the difference in intermediate noise between cotranscribed and uncoupled configurations is greater (typically much greater) than the covariance; noise is much higher in the uncoupled configuration than in the cotranscribed configuration.

Redundant metabolic step

The metabolic product of a redundant metabolic step has lower noise in the uncoupled configuration:

$$\Delta v_P = -v_{AB} \frac{2H_{AP}H_{BP}\left[\tau_{mRNA}\tau_{util} + \tau_{protein}(\tau_{mRNA} + \tau_{util})\right]}{(\tau_{mRNA} + \tau_{util})(\tau_{protein} + \tau_{util})} \tag{S2.4}$$

where τ_{util} is the timescale of product utilization by downstream metabolic processes and the rest of the variables defined as above. Using the same approximations of half lives, we arrive at the simplified relationship

$$\Delta v_P \approx -2v_{AB}H_{AP}H_{BP} \tag{S2.5}$$

where H_{AP} and H_{BP} are both positive (increase in either of the enzymes increases product concentration). The uncoupled configuration then has lower noise than the cotranscribed configuration, with the difference depending on the sensitivity of the metabolic product flux ratio to each of the enzymes.

Metabolic branch point

For the metabolic branch point, we have

$$\Delta \nu_s = -\nu_{AB} \frac{2H_{As}H_{Bs}}{H_{ss}\left(H_{ss}-1\right)} \frac{\tau_{mRNA}+\tau_{protein}-H_{ss}\tau_{mRNA}}{\tau_{protein}-H_{ss}\tau_{mRNA}}. \qquad \text{(S2.6)}$$

For mathematical simplicity, we assume it is symmetric, with identical enzyme kinetics and $\langle A\rangle \approx \langle B\rangle$. The influx of substrate is set by parameter k_{in} and the outflux of product via each enzyme is φ. Then $H_{\xi s} = \frac{1}{2-\frac{k_{in}}{\varphi}}$ (with ξ = A or B) and $H_{ss} = -1 - \frac{K_m}{K_m + s}\frac{1}{\frac{k_{in}}{2\varphi}-1}$, where K_m is the Michaelis-Menten constant for the enzymes (which we assume is identical by symmetry). Because the degradation flux ($s/\tau_{protein}$) is small, $k_{in} \approx 2\varphi$ but with k_{in} slightly larger than 2φ so that H_{ss} and $H_{s\xi}$ are negative. With $\tau_{mRNA} << \tau_{protein}$, the relationship simplifies to

$$\Delta \nu_s = -\nu_{AB} \frac{2H_{As}H_{Bs}}{H_{ss}\left(H_{ss}-1\right)}. \qquad \text{(S2.7)}$$

If the enzymes are unsaturated, $s < K_m$ and the difference is relatively small (from large negative H_{ss}). In the saturated regime, $s \gg K_m$; the absolute value of H_{ss} is smaller than values of H_{ss} for the unsaturated enzymes leading to significantly higher substrate noise in the cotranscribed configuration than in the uncoupled configuration. As shown in Text S3, an ultrasensitive switch exists in this regime.

Multiple gene regulators

The noise difference between cotranscribed and uncoupled regulators of a downstream gene with OR logic is

$$\Delta \nu_P = -\nu_{AB} H_{Am_P} H_{Bm_P} \frac{\tau_{mRNA}}{\tau_{mRNA}+\tau_{protein}}. \qquad \text{(S2.8)}$$

Thus the difference is always negative with the type of transcriptional logic assumed here (both regulators being transcriptional activators), with logarithmic gains H_{Am_P} and H_{Bm_P} positive. The same result holds if both regulators are repressors.

For the logarithmic gains that characterize the effect of A and B on m_p, we find:

$$H_{Am_p} = \frac{K_m k_{mp} \langle A \rangle}{\left(K_m + \langle A \rangle + \langle B \rangle\right)\left[K_m k_{mpb} + \left(k_{mp} + k_{mpb}\right)\left(\langle A \rangle + \langle B \rangle\right)\right]} \tag{S2.9}$$

$$H_{Bm_p} = \frac{K_m k_{mp} \langle B \rangle}{\left(K_m + \langle A \rangle + \langle B \rangle\right)\left[K_m k_{mpb} + \left(k_{mp} + k_{mpb}\right)\left(\langle A \rangle + \langle B \rangle\right)\right]} \tag{S2.10}$$

where parameters are defined in Table S3; specifically, K_m defines the half-saturation concentration for downstream gene induction by A and B, k_{mp} is the maximum regulated production rate of downstream $mRNA_p$, and k_{mpb} is the constitutive basal rate. The sensitivity to each regulator ranges between 0 and 1, limiting the largest possible noise difference between cotranscribed and uncoupled configurations.

Physical protein interaction

For physical protein interactions, the complete LNA solution is bulky. In the limit for strong binding between A and B ($k_d \to 0$) and assuming $\langle A \rangle \approx \langle B \rangle$,

$$\Delta v_A = \Delta v_B = -v_{AB} \frac{H_{AB}(\tau_{mRNA} + \tau_{protein})(2\tau_{mRNA} + \tau_{protein})}{(H_{AB}{}^2 - 1)\left[(H_{AB}{}^2 - 1)\tau_{mRNA}{}^2 - 2\tau_{mRNA}\tau_{protein} - \tau_{protein}^2\right]} \tag{S2.11}$$

$$\Delta v_{complex} = -v_{AB} \frac{2\left[(H_{AB} - 2)\tau_{mRNA} - \tau_{protein}\right]}{(2 - 3H_{AB} + H_{AB}{}^2)(H_{AB}\tau_{mRNA} - \tau_{mRNA} - \tau_{protein})} \tag{S2.12}$$

When $H_{AB} = -\frac{\langle A \rangle k_b}{\langle A \rangle k_b + k_{deg}} \approx -1$, $\Delta v_{complex} \approx -v_{AB}\left(\frac{\tau_{mRNA} + \frac{\tau_{protein}}{3}}{2\tau_{mRNA} + \tau_{protein}}\right)$, and because

$\tau_{mRNA} << \tau_{protein}$, we conclude that $\left|\Delta v_{complex}\right| < \frac{v_{AB}}{3}$ (where $\left|\bullet\right|$ denotes absolute value). On the other hand, with the large timescale difference for mRNA and protein we have

$\Delta v_A = \Delta v_B \approx -v_{AB}\frac{H_{AB}}{1-H_{AB}^2} = v_{AB}\frac{\left|H_{AB}\right|}{1-H_{AB}^2}$, which asymptotically approaches positive infinity as $|H_{AB}|$ approaches 1 from below (binding is much faster than protein degradation). Therefore, for strong binding the noise in the monomer is a dominant effect. In the limit of weak binding, the difference between co-transcribed and uncoupled genes is negligible.

Covalent modification

For the covalent modification module, we have (in the following equations, subscript *m* of *H* refers to mRNA species):

$$\Delta v_{A^*} = -v_{AB}\frac{2\left(\tau_{mRNA} + H_{mA}\tau_{mRNA} + \tau_{protein}\right)}{\left(1 + H_{mA}\right)\left(H_{mA}\tau_{mRNA} + \tau_{protein}\right)} \quad \text{(S2.13)}$$

for the modified form of A, and for the unmodified form,

$$\Delta v_A = v_{AB}\frac{2(H_{mA} - 1)(\tau_{mRNA} + H_{mA}\tau_{mRNA} + \tau_{protein})}{(H_{mA} + 1)(H_{mA}\tau_{mRNA} + \tau_{protein})} \quad \text{(S2.14)}$$

where $H_{mA} = \frac{k_p \langle A \rangle \langle B \rangle}{k_p \langle A \rangle \langle B \rangle - k_{tsnA}\langle m \rangle} > 1$. In this expression k_p is the modification rate and k_{tsnA} is the translation rate of protein *A*. τ_{mRNA} is small, resulting in the approximations

$$\Delta v_{A^*} \approx -v_{AB}\frac{2}{H_{mA} + 1} \quad \text{(S2.15)}$$

and

$$\Delta v_A \approx v_{AB} \frac{2(H_{mA} - 1)}{H_{mA} + 1}. \tag{S2.16}$$

We can therefore conclude that $\Delta v_{A*} < 0$ and $\Delta v_A > 0$. Therefore the same-operon configuration decreases noise in unmodified form of A but increases noise in the A^* form.
