# Supplementary material for: Interplay of Gene Expression Noise and Ultrasensitive Dynamics Affects Bacterial Operon Organization
Source: PLoS Comput Biol. 2012 Aug 30;8(8):e1002672. doi: 10.1371/journal.pcbi.1002672 (PMC3431296; doi:10.1371/journal.pcbi.1002672)
Supplement: Text S3 — Analytical determination of ultrasensitive thresholds. (PDF) [file pcbi.1002672.s016.pdf]

Text S3. Analytical determination of ultrasensitive thresholds

To generalize ultrasensitivity results (Figures 3 and S2) beyond the parameters used for stochastic simulations, we show that the steady state concentration of variables with the ultrasensitive switch (black lines in Figures 3 and S2) typically have the characteristic ultrasensitive response (i.e. the logarithmic gain of some flux or molecule, *y*, in response to changes in another flux or molecule, *x*, $\frac{\partial \log y}{\partial \log x} \gg 1$; [43]) for physiologically relevant parameters.

Linear metabolic pathway

For the linear metabolic pathway, we start with the differential equation:

$$\frac{d[I]}{dt} = v_1[A] - v_2[B]\frac{[I]}{K_m+[I]} - k_{deg}[I].$$

See Table S3 for parameter definitions. Holding the precursor constant, the term $\frac{[S]}{K_{m1}+[S]}$ is subsumed into v_1. Solving the temporal steady state for [*I*],

$$[\hat{I}] = \frac{1}{2k_{deg}}\left[v_1[\hat{A}] - v_2[\hat{B}] - k_{deg}K_m + \sqrt{4v_1[\hat{A}]k_{deg}K_m + (k_{deg}K_m - v_1[\hat{A}] + v_2[\hat{B}])^2}\right]. \qquad \text{(S3.1)}$$

To see the steady-state sensitivity to changes in the ratio of *A* to *B* expression, take $[\hat{A}]$ to be constant and find the logarithmic gain with respect to $[\hat{B}]$:

$$\left.\frac{\partial \log[I]}{\partial \log[B]}\right|_{SS} = \frac{[\hat{B}]}{[\hat{I}]}\frac{\partial[\hat{I}]}{\partial[\hat{B}]} = \frac{v_2[\hat{B}]}{\sqrt{4v_1[\hat{A}]k_{deg}K_m + (k_{deg}K_m - v_1[\hat{A}] + v_2[\hat{B}])^2}}. \qquad \text{(S3.2)}$$

When $v_1[\hat{A}] \approx v_2[\hat{B}]$ in the relevant parameter regime (i.e., when k_{deg} is small and enzyme *B* is saturated),

$$\left.\frac{\partial \log[I]}{\partial \log[B]}\right|_{SS} \approx \frac{v_1[\hat{A}]}{\sqrt{4v_1[\hat{A}]k_{deg}K_m}} = \sqrt{\frac{v_1[\hat{A}]}{4k_{deg}K_m}}. \qquad \text{(S3.3)}$$

For typical conditions, the dilution rate (k_{deg}) is much smaller than the enzyme catalytic rate (v_1), so that the sensitivity is greater than 1 when the production flux $v_1[\hat{A}]$ is sufficiently large relative to the Michaelis-Menten constant K_m.

Metabolic branch point

For the metabolic branch point, we first assume for simplicity that the two enzymes at the branch point bind substrate at the same affinity. Then the steady state takes the form:

$$[\hat{S}] = \tfrac{1}{2k_{deg}}\left[k_{in} - v_1[\hat{A}] - v_2[\hat{B}] - k_{deg}K_m + \sqrt{4k_{in}k_{deg}K_m + (k_{deg}K_m + v_1[\hat{A}] + v_2[\hat{B}] - k_{in})^2}\right]. \quad \text{(S3.4)}$$

See Table S3 for definitions of the parameters). This is very similar to the steady state for metabolic intermediate (Equation S3.1). To see the effect of transcriptional coupling, take k_{in} to be analogous to the term $v_1[\hat{A}]$ in Equation S3.1 and the term $v_1[\hat{A}] + v_2[\hat{B}]$ analogous to $v_2[\hat{B}]$ in Equation S3.1; we use the same sensitivity calculation as above to arrive at a sensitivity peak when $k_{in} \approx v_1[\hat{A}] + v_2[\hat{B}]$. On the other hand, if the enzymes fluctuate independently, we take the term $k_{in} - v_1[\hat{A}]$ as analogous to the term $v_1[\hat{A}]$ in Equation S3.1 and $v_2[\hat{B}]$ analogous to $v_2[\hat{B}]$ in Equation S3.1. We then find

$$\left.\frac{\partial \log[S]}{\partial \log[B]}\right|_{SS} = \frac{[\hat{B}]}{[\hat{S}]}\frac{\partial[\hat{S}]}{\partial[\hat{B}]} = \frac{v_2[\hat{B}]}{\sqrt{4k_{in}k_{deg}K_m + (k_{deg}K_m - k_{in} + v_1[\hat{A}] + v_2[\hat{B}])^2}}. \quad \text{(S3.5)}$$

By analogy to the approximation made for Equation S3.3, the sensitivity of S to B reaches a peak only when the term $v_1[\hat{A}]$ is small, and S may not have an ultrasensitive threshold at all in the uncoupled case if $v_1[\hat{A}]$ is sufficiently large.

Covalent modifications and physical protein interactions

The effects of changing protein production rates on the unbound monomer in the physical interaction module have been analyzed previously [32]. The covalent modification module is similar to the physical interaction module with an additional flux representing conversion to the covalently modified form of protein A. Using the mean-field model as in Table S3, with mRNA variables subsumed into protein concentrations $\alpha_A = \frac{k_{tsnA} k_{mA}}{k_{mdeg} k_{deg}}$ and $\alpha_B = \frac{k_{tsnB} k_{mB}}{k_{mdeg} k_{deg}}$, and following Buchler and Louis [32], we find

$$[\hat{A}] = \frac{1}{2}\left[\alpha_A - \alpha_B \left(1 + \frac{k_p}{k_{deg}}\right) - \kappa + \sqrt{4\alpha_A \kappa + \left(\alpha_B \left(1 + \frac{k_p}{k_{deg}}\right) - \alpha_A + \kappa \right)^2} \right] \qquad \text{(S3.6)}$$

where $\kappa = \frac{1}{k_b}\left(k_d + k_p + k_{deg}\right)$. The ultrasensitive regime occurs where $\alpha_A \approx \alpha_B \left(1 + \frac{k_p}{k_{deg}}\right)$ as long as *A* and *B* have reasonable affinity for each other. To see this, take the logarithmic gain of the *A* production flux with respect to *B*:

$$\frac{\partial \log[\hat{A}]}{\partial \log \alpha_B} = \frac{\alpha_B}{[\hat{A}]} \frac{\partial [\hat{A}]}{\partial \alpha_B} = -\alpha_B \left(1 + \frac{k_p}{k_{deg}}\right) \frac{1}{\sqrt{4\alpha_A \kappa + \left(\alpha_B \left(1 + \frac{k_p}{k_{deg}}\right) - \alpha_A + \kappa \right)^2}}. \qquad \text{(S3.7)}$$

Then with $\alpha_A \approx \alpha_B \left(1 + \frac{k_p}{k_{deg}}\right)$, we have

$$\frac{\partial \log[\hat{A}]}{\partial \log \alpha_B} \approx -\frac{\alpha_A}{\sqrt{4\alpha_A \kappa + \kappa^2}}. \qquad \text{(S3.8)}$$

For sufficiently strong binding affinity, the parameter κ is small relative to α_A and the peak sensitivity can take on very high (absolute) values. In this limit,

$$\frac{\partial \log[\hat{A}]}{\partial \log \alpha_B} \approx -\frac{1}{2}\sqrt{\frac{\alpha_A}{\kappa}}. \qquad \text{(S3.9)}$$
