# Supplementary material for: Interplay of Gene Expression Noise and Ultrasensitive Dynamics Affects Bacterial Operon Organization
Source: PLoS Comput Biol. 2012 Aug 30;8(8):e1002672. doi: 10.1371/journal.pcbi.1002672 (PMC3431296; doi:10.1371/journal.pcbi.1002672)
Supplement: Text S4 — Chromosomal proximity of genes does not explain frequencies of metabolic or physical interaction operons. (PDF) [file pcbi.1002672.s017.pdf]

Text S4. Chromosomal proximity of genes does not explain frequencies of metabolic or physical interaction operons

Operons could allow genes with interacting products to be in close physical proximity, which would enable faster interaction once translation is finished [13], or enable horizontal gene transfer between strains [10]. We sought to determine whether operons can be explained by chromosomal proximity of linear metabolic and physically interacting gene pairs in *E. coli*.

We first found the distribution of chromosomal distances between interacting gene pairs, compared to a control where the loci were randomly reassigned to genes in the network (Figure S5). The linear metabolic pairs show no evident difference from the randomized control (Kolmogorov-Smirnov test, $p \approx 0.36$; Figure S5A) suggesting little or no spatial proximity effect for this case. Gene pairs for physically interacting proteins have a bimodal distribution, with one mode evidently representative of non-randomly close proximity and the other representative of random distances (Figure S5B). Removal of same-operon pairs does not eliminate the lower mode (not shown), so some proximity effect is evident for physical interaction.

To test whether operons exist solely to create the bias toward gene proximity, we performed a chromosome randomization that preserves the underlying distance distribution of interacting gene pairs but otherwise randomizes their loci. For each gene pair, we took the locus of one of the genes and randomly selected a distance from this locus for its interaction pair from the naturally occurring distance distribution. The randomly selected distance leaves two potential loci for the interaction pair, one toward the origin and the other toward the terminus of the chromosome. Both such loci in the *E. coli* chromosome were found, and one of them randomly selected as the locus for the interacting protein. Once gene loci for the interacting pairs were assigned, fractions of interacting gene pairs in the same operon were determined. The process for

the entire set of interactions was repeated 100 times. Each time the distance distributions in interacting pairs were the same as in wild-type chromosome by design of the randomization procedure. Therefore, if operons were to exist solely to create proximity, we would expect the randomized operon frequency to be statistically indistinguishable from the natural frequency in *E. coli*.

For the linear metabolic gene pairs, we found the mean randomized operon frequency with this method to be 0.017 with standard deviation 0.0025, significantly lower than the natural frequency in *E. coli* of 0.037 ($p < 10^{-6}$). Physically interacting gene pairs have a mean frequency 0.24, standard deviation 0.0065, also significantly lower than the natural *E. coli* operon frequency of 0.35 ($p < 10^{-6}$). We conclude that chromosomal proximity cannot explain *E. coli* operon frequencies.
